# Genetic Dissection of the *Canq1* Locus Governing Variation in Extent of the Collateral Circulation

**DOI:** 10.1371/journal.pone.0031910

**Published:** 2012-03-06

**Authors:** Shiliang Wang, Hua Zhang, Tim Wiltshire, Robert Sealock, James E. Faber

**Affiliations:** 1 Department of Physiology, University of North Carolina at Chapel Hill, Chapel Hill, North Carolina, United States of America; 2 Department of Genetics, University of North Carolina at Chapel Hill, Chapel Hill, North Carolina, United States of America; 3 McAllister Heart Institute, University of North Carolina at Chapel Hill, Chapel Hill, North Carolina, United States of America; University Medical Center Utrecht, The Netherlands

## Abstract

**Background:**

Native (pre-existing) collaterals are arteriole-to-arteriole anastomoses that interconnect adjacent arterial trees and serve as endogenous bypass vessels that limit tissue injury in ischemic stroke, myocardial infarction, coronary and peripheral artery disease. Their extent (number and diameter) varies widely among mouse strains and healthy humans. We previously identified a major quantitative trait locus on chromosome 7 (*Canq1*, LOD = 29) responsible for 37% of the heritable variation in collateral extent between C57BL/6 and BALB/c mice. We sought to identify candidate genes in *Canq1* responsible for collateral variation in the cerebral pial circulation, a tissue whose strain-dependent variation is shared by similar variation in other tissues.

**Methods and Findings:**

Collateral extent was intermediate in a recombinant inbred line that splits *Canq1* between the C57BL/6 and BALB/c strains. Phenotyping and SNP-mapping of an expanded panel of twenty-one informative inbred strains narrowed the *Canq1* locus, and genome-wide linkage analysis of a SWRxSJL-F2 cross confirmed its haplotype structure. Collateral extent, infarct volume after cerebral artery occlusion, bleeding time, and re-bleeding time did not differ in knockout mice for two vascular-related genes located in *Canq1*, *IL4ra* and *Itgal*. Transcript abundance of 6 out of 116 genes within the 95% confidence interval of *Canq1* were differentially expressed >2-fold (p-value<0.05÷150) in the cortical *pia mater* from C57BL/6 and BALB/c embryos at E14.5, E16.5 and E18.5 time-points that span the period of collateral formation.

**Conclusions:**

These findings refine the *Canq1* locus and identify several genes as high-priority candidates important in specifying native collateral formation and its wide variation.

## Introduction

Ischemic stroke, myocardial infarction and atherosclerotic disease of arteries supplying the brain, heart and lower extremities are leading causes of morbidity and mortality. Recently, variation in the density and diameter (extent) of the native pre-existing collaterals present in a tissue have become recognized as important determinants of the wide variation in severity of tissue injury caused by these diseases [1]–[4]. Collaterals are arteriole-to-arteriole anastomoses that are present in most tissues and cross-connect a small fraction of distal-most arterioles of adjacent arterial trees. After acute obstruction of an arterial trunk, collateral extent dictates the amount of retrograde perfusion from adjacent trees thus severity of ischemic injury. With chronic obstruction, native collaterals undergo anatomic lumen enlargement, a process termed collateral remodeling or arteriogenesis that requires days-to-weeks to reach completion, resulting in an increase in conductance of the collateral network [5].

Surprisingly, native collateral extent differs widely among “healthy” individuals, ie, those free of stenosis of arteries supplying the heart or brain. Thus coronary collateral flow (ie, CFI_p_) varies more than 10-fold in individuals without coronary artery disease [4], [6]. Likewise, large differences in collateral-dependent cerebral blood flow are evident in individuals after sudden embolic stroke [2], [3], [7]. Moreover, direct measurement of native collateral extent shows that it varies widely among healthy inbred mouse strains [8]–[10]. Variation in healthy humans and mice can be attributed to genetic differences affecting mechanisms that control formation of the collateral circulation, which in mouse occurs late in gestation [11], [12], and to differences in environmental influences and cardiovascular risk factors that recent studies are finding affect persistence of these vessels in adults [13], [14].

We previously identified a prominent QTL on chromosome 7 (*Canq1*, LOD = 29) responsible for 37% of the heritable variation in collateral extent (as well as collateral remodeling) in the cerebral circulation of C57BL/6 and BALB/c mice [15]—strains which exhibit the widest difference among 15 strains examined [9]. Moreover, these same 15 strains had an equally wide variation in infarct volume after middle cerebral artery (MCA) occlusion that was tightly and inversely correlated with collateral density and diameter, thus establishing a strong causal relationship between severity of stroke and collateral extent [9], [16]. Three other loci were responsible for an additional 35% of the variation in collateral extent [15]. Importantly, genetic-dependent differences in collateral extent in the cerebral circulation are shared by similar variation in other tissues, at least in mice [8], [10], [17], [18]. Consistent with this, *Canq1* maps to the same location on chromosome 7 as recently reported QTL linked to hindlimb ischemia (*LSq1*) [19] and cerebral infarct volume (*Civq1*) [16]. Thus, our findings [15] identify the physiological substrate, variation in collateral extent that underlies these QTL.

Efficient Mixed-model Association Mapping with high-density SNPs for the above 15 strains allowed us to narrow *Canq1* to a region (“EMMA region”) [15]. In the present study we sought to further refine this locus and the candidate genes potentially responsible for collateral variation. Identification of the causal genetic element(s) will reveal key signaling pathways controlling collateral formation—about which little is known [1], [12], [17]. Moreover, if subsequently confirmed in humans, this information will aid patient stratification, clinical decision making, and the development of collaterogenic therapies. To this end, we strengthened our association mapping over *Canq1* using additional inbred strains, performed linkage analysis of a second genetic mapping population, measured collateral extent and infarct volume in mice genetically deficient for several candidate genes, and examined expression of 116 genes spanning the EMMA region and *Canq1* in the pial circulation of mouse embryos during the period when the collateral circulation forms.

## Materials and Methods

### Animals

Mouse strains (male, ∼10 weeks-old): LP/J, NON/ShiLtJ (NON), LEWES/EiJ, 129X1/SvJ (129X1), CAST/EiJ, C57BL/6J (B6), BALB/cByJ (Bc), *Itgal^−/−^* (#005257, B6 background), *Il4^−/−^* (#002518, B6 background), *Il4ra^−/−^* (#003514, Bc background) and CXB11.HiAJ (CxB11) were obtained from Jackson Laboratories. PWD were a gift of Dr. Fernando Pardo-Manuel, UNC. F1 progeny obtained from reciprocal mating of SWR/J (SWR) and SJL/J (SJL) were mated to produce a 10-week old F2 reciprocal population. This study was approved by the University of North Carolina's Institutional Animal Use and Care Committee and was performed in accordance with the National Institutes of Health guidelines.

### Phenotyping

Mice were phenotyped for collateral number, diameter and cerebral artery tree territories as described previously [15] (Materials and Methods S1).

### Association Mapping

As detailed previously [15], the EMMA algorithm [20], [21] was applied in the R statistical package to collateral number, obtained from 21 inbred strains comprised of 15 previously reported strains [9] plus 6 newly phenotyped strains. Known and imputed dense SNP data were downloaded from http://phenome.jax.org/db/q?rtn=snps/download and http://compgen.unc.edu/wp/ and pooled. The kinship matrix (pair-wise relatedness) among the 21 inbred strains was calculated using the dense SNP-set within the *Canq1* region, and modeled as random effects [22]. Each SNP within *Canq1* was modeled as a fixed effect. After the mixed model was fitted, an F-test was conducted and a p-value obtained at each SNP location.

### DNA Isolation, Genotyping and Linkage Analysis

Tail genomic DNA from SWRxSJL-F2 mice (n = 123) was genotyped using the 377-SNP GoldenGate genotyping array (Illumina, San Diego, CA). SNP positions were obtained from Build 37.1 of the NCBI SNP database. There are 324 informative markers on 19 autosomes in this array, including 29 on Chr 7 with 6 between 121.5 and 144.5 thus 1 every ∼4 Mb. Collateral number was subjected to linkage analysis using the single QTL model in the R statistical package [15]. Threshold for significant QTL was defined as p = 0.05.

### Bleeding and Re-bleeding Assays

Bleeding (thrombosis) and re-bleeding (thrombolysis) assays were similar to previous methods [23]. Under 1.2% isoflurane anesthesia supplemented with oxygen and with rectal temperature maintained at 37°C, the tail was pre-warmed for 5 min in 50 mL of 37°C saline. A 5 mm length from the tip of the tail was amputated with a fresh sterile #11 scalpel blade, and the tail was immediately returned to the saline. The cut surface was positioned 10 mm below the ventral surface of the body. Bleeding time was the time between the beginning and cessation of bleeding. Re-bleeding time was the time between cessation and resumption of bleeding.

### Middle Cerebral Artery Occlusion and Measurement of Infarct Volume

These were done as detailed previously [15] (Materials and Methods S1).

### Gene Expression

B6 and Bc breeders were paired at 10–12 weeks-age. The presence of a vaginal plug the morning after pairing was designated as 0.5 days post-coitus (E0.5). Embryos were collected at E14.5, E16.5 and E18.5 under deep anesthesia (ketamine+xylazine, 100+15 mg/kg) and staged according to crown-to-rump length. Brains were quickly removed into RNAlater® (Sigma-Aldrich Corp, St. Louis, MO) and stored at −20°C. Approximately 24 hours later the *pia mater* containing the pial circulation was peeled from the dorsal cerebral cortex of both hemispheres under a stereomicroscope and stored in RNAlater® at −20°C. Pia from 8–10 embryos of each strain were pooled for each RNA sample (≥2 litters per pool). Three pooled RNA samples were prepared for each strain and time-point (18 samples; ∼100 embryos/strain). Samples were thawed in RNAlater and homogenized (TH, Omni International, Marietta, GA) in Trizol Reagent (Invitrogen, Carlsbad, CA). Total RNA was purified using the RNeasy Micro Kit according to the manufacturer (Qiagen, Valencia, CA). RNA concentration and quality were determined by NanoDrop 1000 (Thermo Scientific, Wilmington, DE) and Bioanalyzer 2100 (Agilent, Foster City, CA), respectively. Measurement of transcript number was conducted for 139 selected genes and 11 splice variants by the genomics facility at UNC using NanoString custom-synthesized probes (NanoString, Seattle, WA) [24], [25]. Transcript number for each gene was normalized to the mean of 6 housekeeping genes: *Gapdh*, *βactin*, *Tubb5*, *Hprt1*, *Ppia* and *Tbp*.

For quantitative RT-PCR, reverse transcription was performed with SuperScript™ First-Strand Synthesis System (Invitrogen, # 11904-018) following the manufacturer's instructions. Amplification was achieved with SYBR® Green JumpStart™ Taq ReadyMix™ (Sigma, #S4438) on a Rotor-Gene 3000 (Corbett Life Science). Samples were analyzed in triplicate and values averaged. Primer sequences are listed in **Table S1**. Data were analyzed using the Relative Expression Software Tool 2009 (version 2.0.13) and calculations as described [26].

### Statistical Analysis

Values are mean±SEM. Significance was p<0.05 unless stated otherwise. ANOVA and Student's *t*-tests were used as indicated in the figures and tables. For analysis of expression: (1) transcript numbers for strain and embryonic time-point were subjected to 2-way ANOVA, with Bonferroni adjustment of p-values for multiple comparisons (p-values÷150, for 150 transcripts). (2) ANOVA p-values were also derived after correction with the Bonferroni inequality for pre-planned *post-hoc* tests for 2 strains and 3 time points. (3) Student's *t*-tests were used to test effect of strain independent of time, with and without Bonferroni adjustment (p-values÷150). Statistical treatment of association mapping data was as described [15].

## Results

### Collateral Number in CXB11.HiAJ RIL Verifies *Canq1* on Chromosome 7

**Figure 1:**
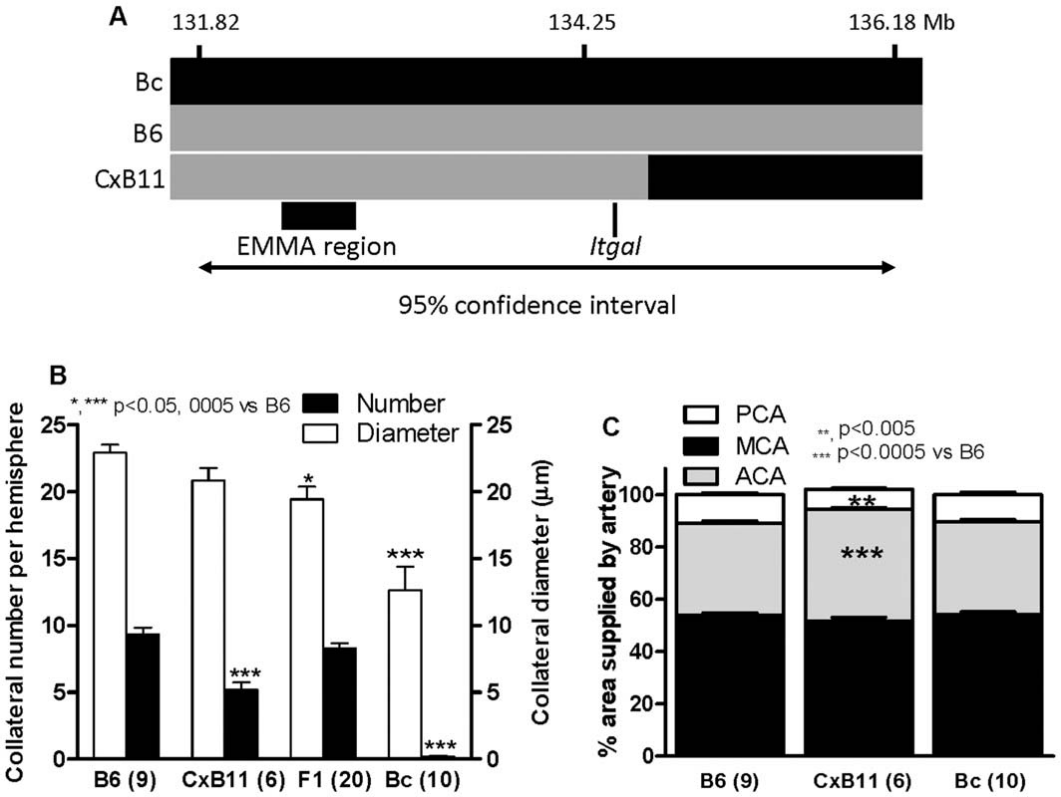
Collateral extent in CXB11.HiAJ (CxB11) confirms importance of *Canq1*. **A**, Inheritance patterns of *Cang1* on chromosome 7 in CxB11 recombinant inbred line derived from BALB/c (Bc)×C57BL/6 (B6). Y axis, SNP positions (Build 37). CxB11 inherits from B6 the segment (B6, grey; Bc, black) that includes the EMMA region and extends through *Itgal*. **B**, Collateral number is intermediate between B6 and Bc in CxB11 and significantly different from B6xBc-F1. **C**, Territory of cerebral cortex supplied by the ACA, MCA, and PCA trees. B6 and Bc are identical (confirms Zhang et al. [9]), while CxB11 has smaller MCA and PCA and larger ACA territory than B6. These differences do not correlate with variation in collateral number or diameter in these or 15 other strains [9]. Numbers of mice given in parentheses in this and other figures.

The CxB recombinant inbred lines (RILs) originated from Bc and B6 parentals [27]. Among them, CxB11.HiAJ (CxB11) is particularly relevant. Given the unique mosaic genetic structure for each RIL, we hypothesized that if *Canq1* in general, and the “EMMA region” [15] in particular, are determinants of collateral extent, an RIL that inherits this locus from either B6 (abundant large-diameter collaterals) or Bc (sparse small-diameter collaterals) [8]–[10], [15] will exhibit traits dominated by the parental phenotype. CxB11 splits *Canq1* such that the centromeric half of *Canq1* and the EMMA region are from B6 (**Figure 1A**). These results confirm our previous findings [15] showing the importance of *Canq1* and the EMMA region for specifying collateral extent.

Because area (“size”) of the MCA, ACA, and PCA trees varies with genetic background and could contribute to variation in collateral extent [9], we measured the percentage of cortical territory supplied by each tree. Values for CxB11 (Figure 1C) fall within the range of differences that have no correlation with variation in collateral extent; also, the parental strains are identical as reported previously [9]. Thus, variation in tree size cannot explain the CxB11 results.

### Additional Inbred Strains Strengthen and Refine the EMMA Region

**Figure 2:**
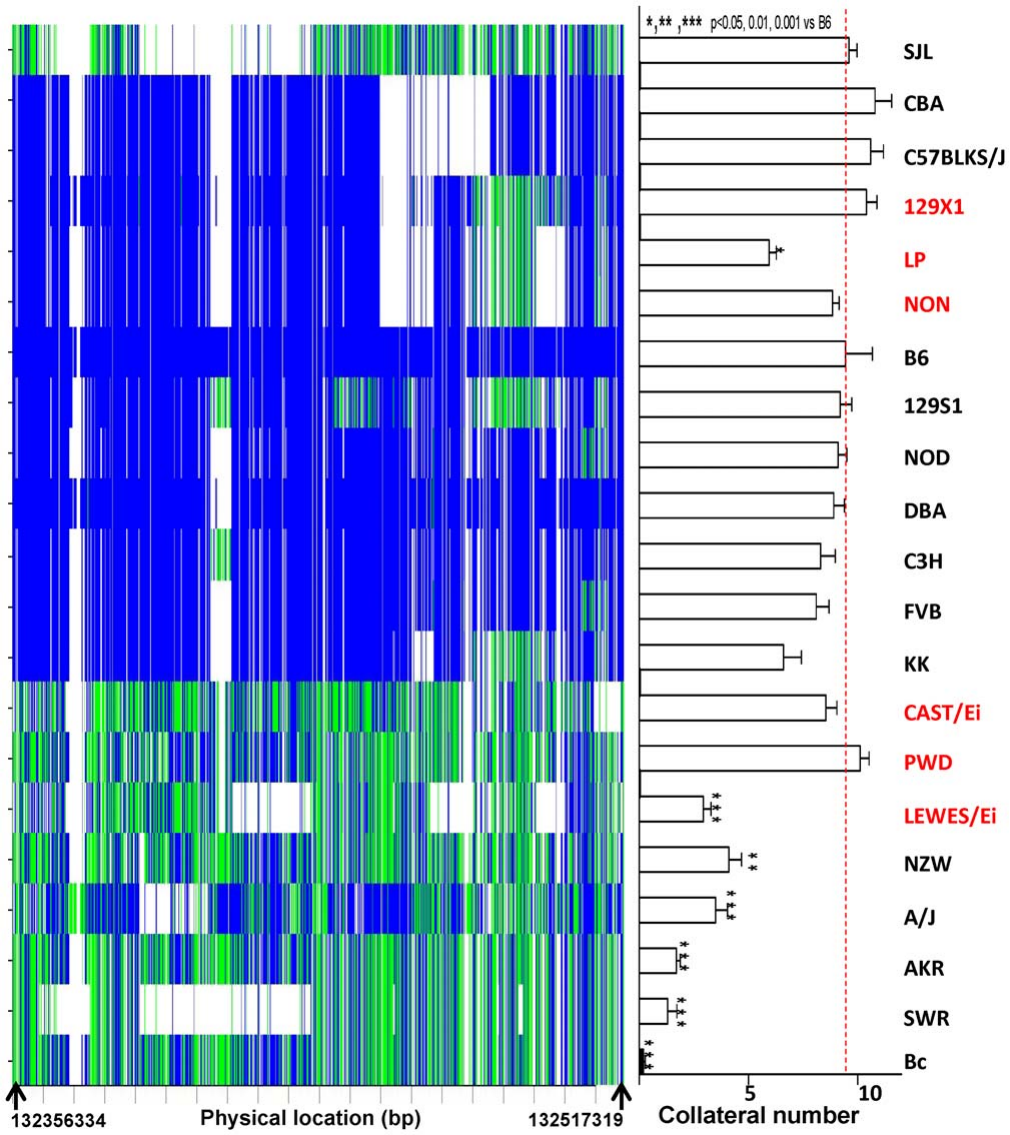
Collateral number for 21 inbred strains and heatmap of their SNPs within the EMMA region. **Left**, Heatmap of known and imputed SNPs for 21 strains in EMMA region (blue, SNP same as B6; green, different from B6; blank, genotype unknown). **Right**, Collateral number per hemisphere for 21 strains. Names in red denote newly phenotyped strains, black names are strains from Zhang et al. [9]. N = 8–10/strain. Dashed line, reference to B6.

EMMA mapping is more efficient computationally and accounts better for population structure embedded in inbred strains, when compared to traditional association mapping algorithms [21]. We previously applied this algorithm to 15 inbred strains and obtained a most-significant 172 kb region (EMMA-A, p = 2.2×10^−5^) and a second-most significant region (EMMA-B, 290 kb, p = 4.2×10^−4^) [15]. To strengthen this analysis, we first generated a heatmap of the known and imputed SNPs within EMMA-A for 74 inbred strains (**Figure S1**). The 15 strains lowest on the y-axis are those used previously [15]. The lowest 5 have significantly fewer collaterals than B6. Among the 15 strains, 9 out of 10 with high collateral number exhibit a DNA haplotype-like block similar to B6 (**Figure 2**, blue), whereas all 5 strains with low collateral number exhibit a haplotype-like block similar to Bc (Figure 2, mostly green). The exception is SJL/J (discussed below). To test the robustness of the EMMA-A region for variation in collateral number, we chose the 6 most informative additional inbred strains from the 74 lines (denoted red in Figure 2): 3 strains with haplotype structures predicting high (129X1, LP, NON) and low (CAST/Ei, PWD, LEWES/Ei) collateral number. Collateral numbers in 4 of these strains fit the prediction from their haplotype structures (**Figure S2**). The remaining two are wild-derived strains that have more complex ancestry and haplotype structures.

**Figure 3:**
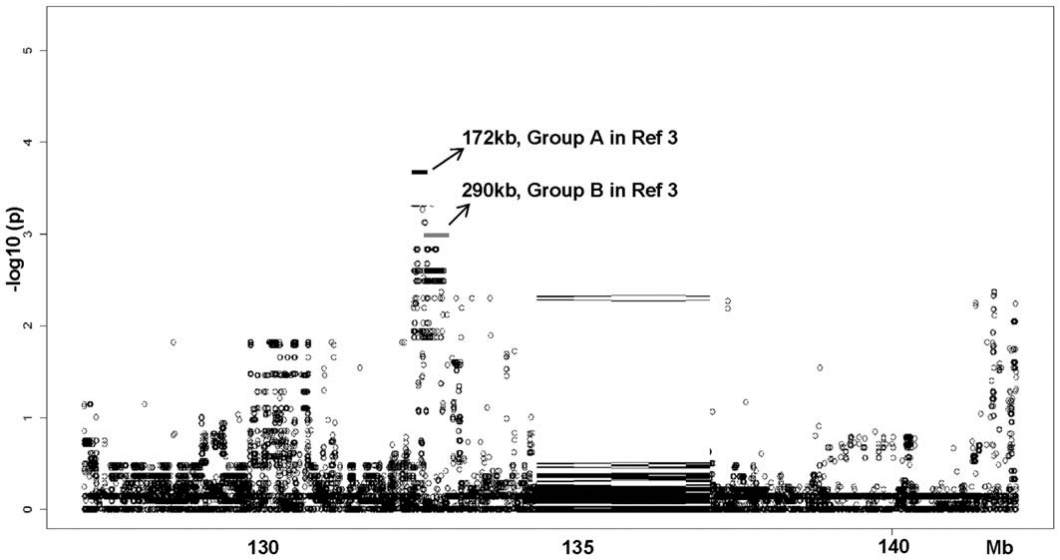
Six more strains strengthen and narrow the EMMA interval. The EMMA algorithm was applied to collateral number for the 21 strain data set, ie, 6 new strains (red strains in Figure 2) plus 15 from Zhang et al. [9]. ∼49,000 high quality imputed SNPs under the *Canq1* peak were tested. The previous highly significant region was strengthened and narrowed (p<9×10^−6^, 4 SNPs). Black bars indicate the most (Group A) and second most (Group B) significant regions in the previous study [15]. Mapping allowed fewer than three SNPs with missing genotypes.

An important consideration is whether the EMMA-A peak shifts position or if the significant SNPs change their p-values from our previous findings [15] after the additional 6 strains are remapped with the original 15. **Figure 3** shows that EMMA-A did not change position, that the previous EMMA-A peak was narrowed further, and that the new peak acquired increased significance for the same SNPs (p = 9×10^−6^).

The kinship matrix used to account for population structure will vary with use of different SNPs as new strains are considered [22]. Furthermore, we previously did not allow missing genotypes to avoid a decrease in statistical power [15]. We thus examined the effect of several parameters on our new mapping results by: allowing fewer than 3 missing genotypes in the strains; using only informative SNPs; excluding wild-derived strains. Irrespective of these mapping parameters, 4 SNPs always had the most significant p-values (rs32978185, rs32978627, rs32973294, and rs32973297; 132.502–132.504 Mb; **Figures S3, S4, S5, S6, S7**).

### Genome-wide QTL Mapping of SWRxSJL-F2 Shows Absence of *Canq1*

**Figure 4:**
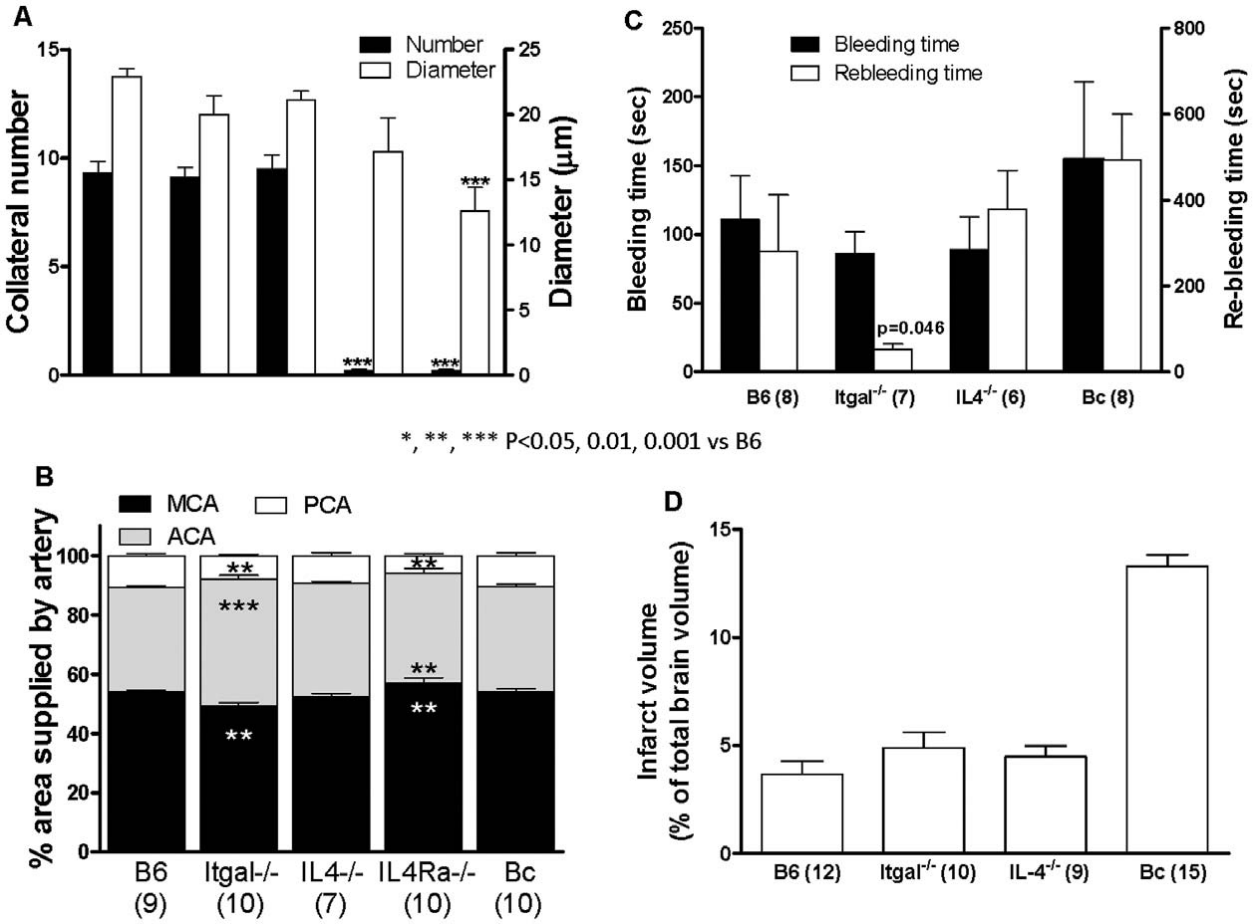
Genome-wide mapping of collateral number in 123 SWRxSJL-F2 mice. **Top panel**, Collateral number per hemisphere among 15 strains of mice (from Figure 2). **Middle panel**, Green = known and high-quality imputed (p>0.90) SNPs common to both strains and BALB/c. White, known and low-quality SNPs different between the two strains, including two common regions with “no useful data”, per JAX website (red). **Lower panel**, LOD profiling using single QTL model. Locations of genotyping SNPs are shown as ticks on the abscissa. 95% confidence level (dashed line) was estimated using 1000 permutations. Insert shows the range of collateral number between the MCA and ACA trees per hemisphere on the abscissa (bin range = 3–14), and number of mice having a given collateral number bin on the ordinate (range 2–23).

Strains SJL and SWR have identical Bc-like haplotype structures in the EMMA regions, but SJL averages 9.6Á±0.4 collaterals per hemisphere while SWR averages 1.3±0.4 (**Figure 4**). To test the validity of the EMMA regions and possibly identify additional QTL, we created a SWRxSJL-F2 cross and performed genome-wide LOD score profiling of collateral number-by-genotype. As predicted, we found no QTL on Chr 7 (Figure 4). We also found no QTL elsewhere in the genome. We did not measure collateral diameter because 1) of the time required to obtain average collateral diameter for each of the 123 F2 mice, 2) we have shown that variation in collateral number and diameter map to the same *Canq1* locus [15], 3) collateral diameter has a much smaller range of variation than number, thus lower LOD score [15], and 4) no significant QTL was found for collateral number (Figure 4).

### Collateral Extent, Hemostasis and Infarct Volume in *Il4ra^−/−^*, *Il4^−/−^* and *Itgal^−/−^* Do Not Support these Genes as Candidates for *Canq1*

**Figure 5:**
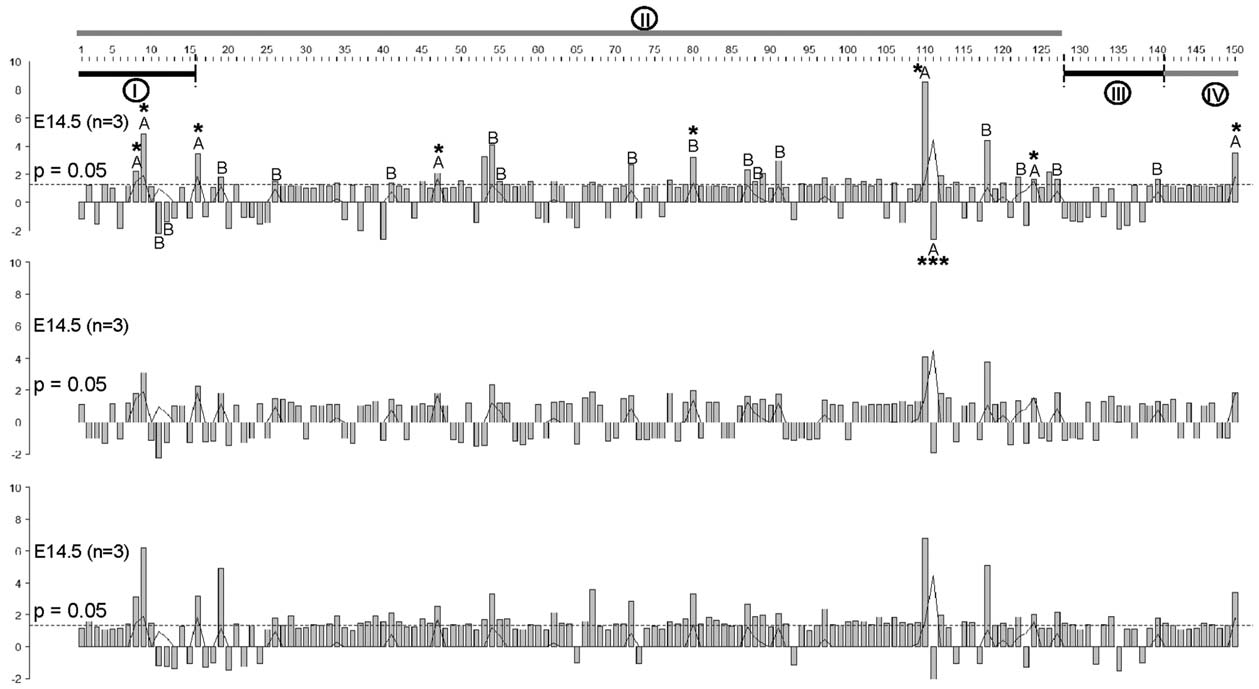
Collateral extent is not altered in *Itgal, IL4 or IL4ra* deficient mice relative to host strains. **A,** *Itgal^−/−^* and *Il4^−/−^* mice are B6 while *Il4ra^−/^* ^−^ (receptor alpha) are Bc background. Number and diameter are not significantly different among *Itgal^−/−^*, *Il4 ^−/−^* and B6, nor between *Il4ra^−/−^* and Bc (t-tests). **B,** Territory (size) of ACA, MCA, and PCA trees are same in B6 and Bc but differ in *Itgal^−/−^* and *Il4ra^−/^* ^−^, confirming dissociation of collateral and tree territory phenotypes among inbred mouse strains [9]. **C,D,** Bleeding and infarct volume of knockouts are not different from B6; re-bleeding in *Il4^−/−^ is* not different from B6 but shorter in *Itgal^−/−^* (*t*-tests).

Collateral extent in the adult is determined by mechanisms that specify their formation in the embryo. Recent studies indicate that *Vegfa, Clic4, Flk1, Adam10 and Adam17* are important in this process [11], [12], [17], [18]. However, none of these genes nor others known to impact their expression or signaling pathways are located in EMMA-B, with the exception of *IL4ra* (interleukin 4 receptor alpha chain). Expression of *Il4ra* is lower in Bc compared to B6 (see below). Moreover, IL4rα is present on endothelial cells (and other cell types) where it dimerizes with IL13rα or γc to mediate responses to IL4 and IL13. These include increased expression of VEGF-A; furthermore, hypoxia-induced VEGF-A expression and angiogenesis are reduced in *Il4^−/−^* mice [28]–[30]. We thus examined *Il4^−/−^* (B6 background) and *Il4ra^−/−^* (only available on Bc background) mice. Collateral extent in *Il4^−/−^* mice (9.5 collaterals, 21 um diameter) was not different from B6 (9.3 and 23 um) (**Figure 5**). Likewise, collateral extent in *Il4ra^−/−^* mice (0.2 collaterals, 17 um) was similar to Bc (0.2 and 13 um). These results do not support involvement of *IL4ra* in variation of collateral extent. Knockout mice are not available for the other genes (see below) in EMMA-B.

Given the limitations of any mapping algorithm, it is possible that a genetic element(s) outside of EMMA-B could underlie *Canq1*. Several findings identify *Itgal* (integrin α-L chain, CD11a) as a candidate. *Itgal* is located at 134 Mb near the peak of *Canq1*. Itgal dimerizes with integrin β2-chain (CD18) to form LFA-1 which is present on platelets and most leukocyte types and binds ICAM1 on endothelial cells, leading to firm adhesion and transmigration [31], [32]. Keum and Marchuk identified *Itgal* as a candidate gene within the *Civq1* QTL for infarct volume measured 24 hours after permanent MCA occlusion in a B6xBc-F2 population [16], and *Itgal^−/−^* mice have smaller infarct volumes 24 hours after 1-hour transient MCA occlusion model [32]. However, *Itgal^−/−^* mice had the same collateral extent as B6 controls (Figure 5). Consistent with this, infarct volume 24 hours after permanent MCA occlusion was not different in *Itgal^−/−^* mice (Figure 5). Infarct volume was also not affected in *Il4^−/−^* mice (B6 background; Figure 5), in agreement with no effect on collateral extent. These findings do not support *Itgal or Il4ra* as candidate genes for *Canq1* [15] *or Civq1* [16].

Variation in MCA tree size among strains is also a determinant of variation in infarct volume after permanent [9] or transient [33] MCA occlusion. MCA tree size was smaller in *Itgal^−/−^* mice (Figure 5), which may contribute to their smaller infarct volume after transient MCA occlusion [32].

The involvement of other factors besides collateral extent in determining severity of brain ischemia after acute occlusion could underlie previous [31] findings. Leukocyte/platelet adhesion and hemostasis have complex influences on infarct volume, especially after transient occlusion, through effects on thrombosis and thrombolysis (ie, hemostasis) and inflammation [33], [34]. Moreover, thrombosis and thrombolysis vary with genetic background [23]. Because these mechanisms could be affected in *Itgal^−/−^* and *IL4^−/−^* mice, we measured bleeding and re-bleeding time to assay thrombosis and thrombolysis, respectively. No differences were observed among knockouts and B6 controls (Figure 5), with the exception of shorter time to re-bleeding in *Itgal^−/−^* mice (discussed below). Depending on models and severity of stroke, increased thrombolysis can either reduce no-reflow and lessen infarct volume, or increase petechial hemorrhage and no-reflow and increase infarct volume [33], [34]. *IL4ra^−/−^* mice were not analyzed for bleeding and re-bleeding because of their cost, because *IL4^−/−^* showed no differences in these assays, and because the data in Panels A and B of Figure 5, where both *IL4^−/−^* and *IL4ra^−/−^* mice were studied, provided no support for these genes in the difference in collateral extent in mice.

We also studied hindlimb ischemia [10] in *Il4^−/−^*, *Itgal^−/−^* and their B6 background strain (**Figure S8**) and obtained results in agreement with the above cerebral studies for lack of effect on native collateral extent in a second tissue—skeletal muscle. Although not the subject of this study, recovery of blood flow was less in *Il4^−/−^* mice, suggesting reduced collateral remodeling (see Figure S8 for relevant references). Blood differential cell count **(Table S2**) identified that *Itgal^−/−^* (but not *IL4^−/−^*) mice have reduced platelets compared to their B6 background strain, which may contribute to their shorter re-bleeding time (Figure 5); they also have increased total leukocytes, lymphocytes, granulocytes and monocytes, which has been reported previously (see Table S2 for corroborating references).

### Expression of Genes within *Canq1*

A plausible explanation for the strong effect of the *Canq1* locus is that a gene(s) with strong influence on collateral extent is differentially expressed between B6 and Bc. To begin to identify possible candidates, we measured transcript levels of genes within *Canq1* containing SNPs in their coding and 2 Kb flanking regions from the pial circulation of B6 and Bc embryos at E14.5, E16.5 and E18.5. Collaterals begin to form as a plexus between the crowns of the cerebral artery trees at E14.5 and reach peak density by E18.5 [11], [12]. Bc embryos form ∼90% fewer collaterals than B6, resulting in a similar large difference in collateral number and diameter in the adult [12]. We thus reasoned that expression would need to be examined at the time collaterals form. Four groups of genes were examined: all genes and splice variants annotated within EMMA-A/B (group I, 10 genes and 5 splice variants; **Table S3**); genes with SNP variation between B6 and Bc strains in coding or regulatory regions (ie, 2 kb 5′ and 3′ to coding) within the 95% CI of *Canq1* (group II, 106 genes and 6 splice variants); selected genes located elsewhere in the genome that are angiogenesis-related (group III, 13 genes) and proliferation-related (group IV, 10 genes).

**Figure 6:**
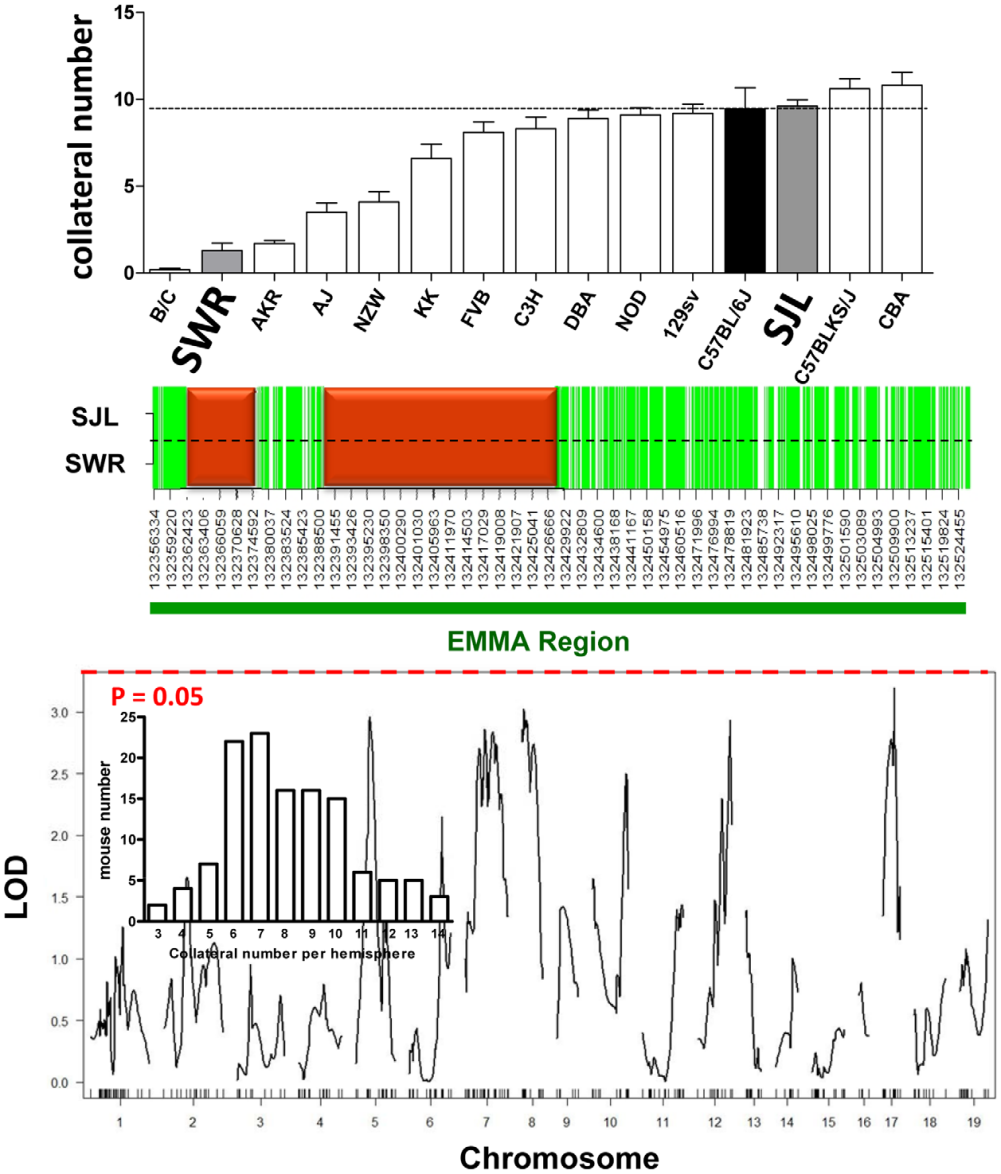
Fold changes between BALB/c and C57BL/6 in mRNA levels of genes within *Canq1* and selected angiogenic- and proliferation/aging-related genes at embryonic day E14.5, E16.5 and E18.5. The numbers 1–150 at top denote gene numbers in Table 1 and Table S2. Y-axis, fold changes; Bc vs B6 if positive, B6 vs Bc if negative. Genes 1–127 located in 95% CI of *Canq1* are arranged in order of physical location. **I**, genes within the EMMA region (132.356–132.528 Mb); **II**, genes in 95% CI of *Canq1*; **III**, angiogenic-related genes; **IV**, proliferation-related genes. Black curve, negative logarithm Bonferroni-adjusted p-values (p-value÷150) for strain effect in 2-way ANOVA; *p<0.05, **p<0.01, ***p<0.001 (÷150). Letters above bars denote significance derived from p-values for 2-way ANOVA for strain after Bonferroni inequality corrected *post-hoc* tests; A, p.strain<0.001, B, p.strain<0.01.

Among those examined, 1 gene in EMMA-B (*Nsmce1*), 6 genes located within the 95% CI of *Canq1* (*Pycard*, *Inpp5f*, *Tbx6*, 3 “Riken genes”) and one located elsewhere (*Tert*, telomerase) had adjusted p-values less than 0.05 for a difference between strains at all 3 time-points using the most stringent analysis, ie, 2-way ANOVA plus Bonferroni adjusted p-value÷150 (star-labeled bars in **Figure 6**; **Table 1**; Table S3). All but *Inpp5f* were more than 2-fold different between B6 and Bc. *Nsmce1* has three splicing isoforms, with two expressing 2.5-to-7-fold higher levels in Bc. *Ino80e* was differentially expressed >2-fold at all time-points, but not significant (although significant with other tests, see below). *Il4ra and Il21r* in EMMA-B were only significant by *t*-test (**Tables S4** and **S5**).

**Table 1:** Genes having differential expression either between strains or among different embryonic days.

Gene* number	Name	Description	Gene† Group	Start	Fold‡ E14.5	Fold‡ E16.5	Fold‡ E18.5	Bonadj§ strain	Bonadj§ Eday
111	Pycard	PYD and CARD domain containing Gene	II	135135617	−2.29	−1.67	−1.79	0.0003	0.0595
124	Inpp5f	inositol polyphosphate-5-phosphatase F	II	135754842	1.90	1.69	2.30	0.0036	1.0000
47	Tbx6	T-box 6	II	133924997	2.38	2.09	2.89	0.0055	1.0000
16	D430042O09Rik-204	RIKEN cDNA D430042O09 gene	II	132851390	3.94	2.61	3.65	0.0073	1.0000
9	Nsmce1-003	non-SMC element 1 homolog	I	132611154	5.54	3.58	7.09	0.0084	1.0000
150	telomerase	NM_009354.1	IV	73764438	3.99	2.14	3.87	0.0085	1.0000
8	Nsmce1-002	non-SMC element 1 homolog	I	132611154	2.52	2.05	3.57	0.0122	1.0000
26	Nfatc2ip	nuclear factor calcineurin-dependent 2 interacting protein	II	133526368	1.68	1.69	2.05	0.0125	1.0000
55	Taok2	TAO kinase 2	II	134009192	1.65	1.38	1.97	0.0143	1.0000
110	B230325K18Rik	RIKEN cDNA B230325K18	II	135126593	9.69	4.68	7.75	0.0165	1.0000
91	1700120K04Rik	RIKEN cDNA 1700120K04 gene	II	134747592	3.37	1.99	2.35	0.0177	1.0000
80	9130019O22Rik	RIKEN cDNA E430018J23 gene	II	134525774	3.63	2.28	3.78	0.0190	1.0000
87	1700008J07Rik	non-coding RNA	II	134655683	2.64	1.87	3.05	0.0202	1.0000
120	BC017158	UPF0420 protein C16orf58 homolog	II	135414893	1.56	1.43	1.67	0.0226	1.0000
140	Vegfa188	vascular endothelial growth factor A	III	46153942	1.88	1.45	2.05	0.0236	1.0000
88	Phkg2	phosphorylase kinase, gamma 2	II	134716854	1.70	1.34	2.19	0.0250	1.0000
19	D430042O09Rik-209	RIKEN cDNA D430042O09 gene	II	132851390	2.04	2.09	5.63	0.0284	1.0000
41	Giyd2	GIY-YIG domain containing 2	II	133832982	1.57	1.63	2.40	0.0291	1.0000
54	Ino80e	INO80 complex subunit E	II	133995094	4.64	2.71	3.79	0.0343	1.0000
127	Dock1	dedicator of cytokinesis 1	II	141862370	1.87	2.15	2.47	0.0384	1.0000
122	Tial1	Tia1 cytotoxic granule-associated RNA binding protein-like 1	II	135583291	2.02	1.54	2.12	0.0418	1.0000
109	Fus	malignant liposarcoma gene	II	135110971	1.47	1.49	1.72	0.0428	0.6911
40	Sult1a1	sulfotransferase family 1A, phenol-preferring, member 1	II	133816379	−2.29	1.01	1.76	1.0000	0.0026
129	Angpt2	angiopoietin 2	III	18690263	−1.15	1.14	1.61	1.0000	0.0077
139	Tgfb1	Transforming gr factor β binding protein 1	III	75404869	1.30	1.18	1.32	0.1245	0.0087
37	Nupr1	nuclear protein 1	II	133766763	−1.72	1.21	1.70	1.0000	0.0096
65	Qprt-001	quinolinate phosphoribosyltransferase	II	134250628	−1.55	−1.19	1.10	1.0000	0.0377
20	AC150648.2	GSG1-like	II	133024217	−1.59	−1.28	−1.27	1.0000	0.0448

Expression assay by NanoString nCounter. 3 RNA samples of pia at each time point for each strain, with each sample composing of ≥8 embryos from ≥2 litters (18 samples, ∼200 embryos). Transcript number for each gene normalized to mean transcript number for 6 housekeeping genes: *βactin*, Gapdh, *Tubb5, Hprt1, Ppia, Tbp*.

* Gene number same as in Figure 6 and Table S2.

† **I**, genes in EMMA region; **II**, genes in 95% CI of Chr 7 QTL; **III**, angiogenic-related genes located elsewhere in genome; **IV**, proliferation-related genes located elsewhere in genome.

‡ Fold change for Bc vs B6 if positive and B6 vs Bc if negative.

§ Bonferroni-adjusted p values (p-value÷150, for 150 genes/splice forms assayed) from 2-way ANOVA for 2 strains (20 genes p<0.05) and 3 embryonic days (6 genes p<0.05).

Expression of 6 genes changed with embryonic time but not strain (listed last in Table 1). Changes in two of these, *Angpt2* (angiopoietin-2) and *Tgfb1* (*Ltbp1*), have well-known roles in vascular development. Thus, the expected changes serve as a control for the NanoString assay. In addition, we confirmed results with qRT-PCR [24], [25], using the above RNA samples from Bc embryos (insufficient RNA prevented qRT-PCR for additional genes or for B6 embryos). Fold-expression values at E18.5 relative to E14.5 for *Angpt2*, *Tgfb1* and *Klf4* (normalize to β-actin) were 4.0±0.7, 3.3±0.9 and 0.7±0.1 by NanoString; and 3.0±0.3, 4.0±0.8 and 1.0±0.1 by qRT-PCR (p = 0.26, 0.60 and 0.09 between assays). The stem cell factor *Klf4* was chosen for comparison, based on its expected absence of change in the vasculature at this late time in gestation.

Since our *a priori* hypothesis was that a difference exists between the strains in expression of at least one of the 116 selected *Canq1* genes, a less stringent level of significance than used in genome-wide cDNA array analysis was warranted, ie, 2-way ANOVA with p-value corrected using the Bonferroni inequality for pre-planned comparisons among 2 strains and 3 time-points. Thus for strain differences, 7 genes had corrected p-values less than 0.001 and 15 less than 0.01 (Figure 6, letters above bars). Seven genes also had significant differences over time (Table S3, corrected p<0.05). Table 1 lists 19 of these genes different by strain (“Bonadj strain”; only *BC017158* and *Fus* did not have a corrected p<0.01 by this analysis) and Table S3 lists the remaining 4—*Il4ra*, *Il21r*, *AC133494.1* and *Tgfb1i1*—which had corrected p-values less than 0.01. Since our *a priori* hypothesis was that a strain difference exists in expression of one or more of the 116 *Canq1* genes, independent of time, we also conducted *t*-tests (Tables S4 and S5). Twenty-three genes were significant at p<0.01, and 43 at p<0.05 which is not unexpected given their location in different haplotype blocks.

## Discussion

We recently identified *Canq1* as a primary locus responsible for variation in extent of the collateral circulation in mice [15]. In the present study we sought to refine this locus and identify candidate genes responsible for collateral variation. Collateral density was reduced by approximately 50% in CxB11 RIL mice in which *Canq1* is split between B6 (proximal half) and Bc strains. This suggests that *Canq1* harbors a polymorphism(s) possibly in its proximal half that governs variation in collateral extent. That a greater reduction was not obtained may reflect mosaicism elsewhere in the CxB11 genome (∼60% Bc), including at three other QTL that we found on Chr 1, 3 and 8 with small effects (*Canq-2,-3,-4*, LODs of 5–13) [15]. Since multiple regulatory loci can be linked in *cis* (as well as *trans*), it is also possible that a variant in each half of *Canq1* could be involved—a possibility supported by the cluster of significant SNPs mapped to the far 3′ of *Canq1* (Figure 3, Figures S3, S4, S5, S6, S7). Generation of congenic strains that partition *Canq1* may allow differentiation between these possibilities.

We previously used association mapping in 15 classical inbred strains and narrowed *Canq1* to 170 and 290 kb regions (EMMA-A and-B regions) [15]. To test the robustness of the EMMA-A region for collateral variation and possibly narrow it further, we generated a SNP heatmap for 74 inbred strains and identified the 6 most informative additional inbred strains—3 classical and 3 wild-derived. Association mapping of this 21-strain panel narrowed EMMA-A and increased its statistical significance. While 4 of the 6 new strains had collateral numbers predicted by their haplotype structure, Cast/Ei and PWD were outliers (Figure 2). The unique haplotype structure of these wild-derived strains has discouraged their use in previous mapping studies [35], [36], a finding which our results confirm. However, the EMMA haplotype of the wild-derived LEWES strain fit the predicted collateral number. What might explain this conundrum? Several studies have utilized dense-SNP data from recent re-sequencing efforts (NIEHS and Perlegen) to infer ancestral haplotype origins among the classical inbred strains [35], [36]. Using WSB, PWD and CAST/Ei as reference strains for *Mus Musculus* subspecies *domesticus*, *musculus* and *castaneus*, together with the hidden Markov Model algorithm, Frazer and colleagues [35] found that 11 classical inbred strains shared 68% haplotype origins with *domesticus* (WSB), 6% with *musculus* (PWD), and 3% with *castaneus* (CAST/Ei). In another study, Yang et al [36] found that wild-derived strains, which are presumed to represent different subspecies, have substantial inter-subspecific introgression. Using the 72% of the autosomal and X-chromosomal 100 kb genomic intervals, for which the ancestry of the reference strains is unambiguous, they estimated that the 11 classical inbred strains inherited 92% of their genomes from *Mus domesticus* origin, 3–12% from *musculus* origin and 1–2% from *castaneus* origin. In a recent study, Kirby and co-workers [37] densely genotyped 121,000 SNPs for 94 inbred strains. Inspection of these data shows that LEWES has close ancestral origins with the WSB strain. Based on the above studies, we postulate that CAST/Ei and PWD are outliers in our study because they share minimal ancestral haplotype origins with the classical inbred strains, while LEWES shares major ancestry origin with the classical inbred strains and the predicted collateral number. Importantly, regardless of whether the wild-derived strains were included or excluded in our association mapping, the same significant region was obtained with similar p-values (Figures S3, S4, S5). Moreover, the diverse genetic background used in our mapping was corrected by applying the kinship matrix. For example, the average identical-by-descent between CAST/Ei and the other 20 stains is 0.45 m, and it is 0.82 between B6 and the other 20 strains. In addition to the above, a significant additional finding from the 21-strain analysis is that it suggests that *Canq1* is not only important in controlling collateral formation in the B6 and Bc strains, but also in 19 additional strains and thus in the mouse species itself.

The SJL strain, which has high collateral density yet possesses an EMMA haplotype structure apparently identical to SWR, is also outlier (Figures 2 and 4). Besides the known SNPs that are identical in EMMA-A, ∼400 SNPs imputed with high confidence (>0.9) are identical between SWR and SJL, and the remaining are also the same among the ∼1000 SNPs imputed with lower confidence. What genetic component(s) regulates the variation in native collateral extent between SWR and SJL if no variation exists within EMMA-B? To identify possible additional QTL, plus confirm our previous [15] and current mapping results, we performed genome-wide linkage analysis on a SWRxSJL-F2 cross. No QTL on chromosome 7 was present, a finding congruent with the common haplotype-like block for these strains. These results also strengthen our previous confirmation of *Canq1* using association mapping and chromosome substitution strain analysis [15], and the finding by others [16] of a QTL for cerebral infarct volume (*Civq1*) at the same location as *Canq1*, using an SWRxB6-F2 cross. Our finding of no other QTL could reflect differences in size of the F2 population and/or fold-differences in trait (collateral number) in our SWRxSJL-F2 cross (n = 123 and 6-fold) versus our previous B6xBc-F2 [15] cross (n = 221 and 30-fold). It could also reflect the presence of additional smaller-effect loci, which is supported by the distribution of the collateral number trait (lower panel of Figure 4).

It is noteworthy that A/J, with its low collateral number (and diameter, Figure S2), also has some outlier-like haplotype features (Figure 2). Yet, collateral number and diameter “follow” chromosome 7 by ∼70% using B6→A/J chromosome substitution strain (CSS) analysis [15]. And 7 of the EMMA-B genes do not fit the CSS analysis “in a simple world”, eg, *Gtf3c1* is indistinguishable between A/J and B6 except for 1 SNP, whereas A/J and Bc differ by 166 SNPs (JAX SNP Browser). This would seem to eliminate *Gtf3c1* as a candidate gene, a finding supported by its lack of differential expression by even *t*-test analysis (Table S5). However, compared to the other 20 strains across EMMA-B, A/J appears as a mix. Some genes are identical to B6 while others are identical to Bc, in contrast to the impression from looking at the haplotypes in Figure 2. These considerations emphasize the likelihood that other known (*Canq2-4* [15]) and unknown loci besides *Canq1* are involved.

The most significant EMMA-A region (132.502–132.504 Mb) lacks mRNA annotation (http://uswest.ensembl.org/Mus_musculus/Location/View?r=7:132495610-132513000). This was confirmed using the UCSC genome browser database. Mammalian transcription factor binding elements are also lacking (Transcription Element Search System, TESS, http://www.cbil.upenn.edu/cgi-bin/tess/tess?RQ=WELCOME). EMMA-A and EMMA-B (132.495–132.513 Mb) also lack annotated microRNAs and other nc(non-coding)RNAs (http://www.ncrna.org/). However, 10 genes are annotated in EMMA-B (discussed below), as well as hundreds of expressed sequence tags (ESTs).

Since large-effects QTL like *Canq1* are usually accompanied by changes in expression of the responsible polymorphic gene in the QTL and/or genes within its effector pathway that are located elsewhere in the genome, transcript analysis is a critical tool used to identify candidate genes. We thus measured expression of all 10 of the annotated genes within EMMA-B and 106 genes in the 95% CI of *Canq1* having SNP differences in their coding or regulatory (±2 kb) sequence. RNA was obtained from the *pia mater* of B6 and Bc embryos at E14.5, E16.5 and E18.5. Although the *pia mater* is an approximately 5 µm thick collagen membrane containing dispersed fibroblasts and IB4 lectin-staining hematopoietic-like cells, these are greatly outnumbered by the endothelial cells that compose the arterial trees, pial capillary plexus, and the collateral circulation that forms during this interval [11], [12]. To our knowledge none of the genes in EMMA-B or those in the wider 95% CI of *Canq1* is known to be involved in angiogenesis. Thus, one or more of the differentially (or not, see below) expressed genes may be a novel “collaterogenesis” gene that controls, in *trans*, expression of a gene(s) located elsewhere in the genome that has been shown to alter collateral formation, eg, *Clic4* (Chr4) [18], *Vegfa* [12], [17] (Chr17), *Flk1* [12] (Chr5), *Adam10* [12] (Chr9) and *Adam17* [12] (Chr12).

*Nsmce1*, *Tbx6*, *Ino80e*, *Pycard* and 3 “Riken genes” within the 95% CI of *Canq1* and one located elsewhere (*Tert*, telomerase) were differentially expressed greater than 2-fold at all 3 embryonic time-points at the highest level of significance (p<0.05÷150) (*Ino80e* just missed this level) (Figure 6, Table 1). The EMMA-B gene *Nsmce1* (non-structural maintenance of chromosomes (Smc) element-1; Nse1) was 5-fold greater in Bc than B6 (2^nd^ largest fold-difference among the 150 transcripts assayed). Nsmce1 is part of a multi-protein complex important in telomere maintenance during proliferation, compensation for telomerase deficiency, inhibition of apoptosis, and promotion of cell survival [38]–[42]. These functions may be important in collaterogenesis.

*Tbx6* (T-box-6 transcription factor) is required for mesoderm, somite and left-right body axis specification that involves Wnt/β-catenin and BMP/TGFβ1→Notch/Delta signaling [43], [44]. Cross-talk exists among the Tbox, Wnt/β-catenin, TGFβ1, VEGF and Notch pathways [45]–[47]. Although no information is available regarding interaction between *Tbx6* and *Vegfa*, this is well-established for *Tbx1* [48] and less-so with *Tbx2* [49]. Evidence that VEGF-A/Flk1→Delta/Notch drives collateral formation [12], together with the above findings, identify *Tbx6* as a candidate.

*Ino80e* (regulator of inositol responsive gene expression; Ccdc95) in human is one of the 16 subunits of the INO80 chromatin remodeling complex. INO80 controls transcription of genes that regulate cell proliferation, differentiation and embryonic development, as well as DNA replication, repair and telomerase activity [50], [51]. Thus *Ino80e*, *Nsmce1* and *Jmjd5* (below) are attractive epigenetic candidates capable of regulating multiple genes.

*Pycard* (apoptosis speck-like protein containing a C-terminal caspase recruitment domain; ASC) was the most strongly downregulated gene in Bc with the lowest p-value (0.00004) among the 127 *Canq1* transcripts examined (Table 1). Pycard is an adaptor protein for NLRP3 (nucleotide binding leucine rich repeats-containing protein 3) that is increased by cell-stress stimuli and required for formation of the multi-protein inflammasome complex, which is induced in activated macrophages and causes interleukin secretion, additional macrophage recruitment, and caspase-1-mediated apoptosis [52]–[54].

The Riken ESTs *D430042O09Rik*, *9130019O22Rik* and *B230325K18Rik* may be ESTs for long-ncRNA “genes” [55]–[57]. Although little annotation is available for this relatively new family of RNA genes, databases www.biogps.org and www.nextbio.com/bodyatlas show the following: *D430042O09Rik* is ubiquitously expressed and greater in Bc that B6 tissues, and has conserved homology through *C elegans*; *9130019O22Rik* is ubiquitously expressed at comparable levels in both strains; *B230325K18Rik* is comparable in Bc and B6. If these loci function as lncRNAs they could regulate one or multiple genes involved in collateral formation and maintenance.

*Jmjd5*, located just 3′ to EMMA-B, was not differentially expressed. However, a loss-of-function or gain-of-function variant in a gene could be important yet not be accompanied by a change in expression. *Jmjd5* (jumongi domain-containing protein 5; lysine-specific demethylase 8) is a newly discovered member of the jumongi-C domain-containing proteins that are receiving considerably attention [58]–[60]. Some JmjC proteins are histone demethylases involved in epigenetic regulation. Although nothing is known about mouse Jmjd5 function, human JMJD5 (aka KDM8) is a H3 lysine36 demethylase and transcriptional activator of cyclin A1 that is required for cell cycle progression and is a potential tumor suppressor. Given the rapidly expanding roles for histone demethylases in controlling coordinate expression of multiple genes within a pathway, *Jmjd5* is a candidate gene.

In our previous study [15] we examined all genes in the 95% confidence interval of *Canq1* using Ingenuity Pathway Enrichment Analysis. Thirteen of 20 known pathways showed significance (p<0.05, Benjamini-Hochberg), with cell-cell signaling and immune response pathways showing the greatest enrichment. However, no additional referenced gene connections were identified beyond those discussed above.

We also measured expression of 13 genes that control angiogenesis and 10 involved in endothelial cell proliferation that reside outside of *Canq1*. Only *Tert* (a protein component of telomere reverse transcriptase (telomerase)) was significantly differentially expressed. Increased expression of this chromosome-stabilizing, anti-aging/cell-survival protein [61] in Bc mice may be part of a compensatory pathway activated by deficiencies in an above-mentioned gene(s) that are predisposing to failure to form or loss of nascent collaterals during embryogenesis. Telomere-independent functions have also been recently identified for telormerase [62]. Telomerase is a cofactor in the β-catenin transcriptional complex, facilitating canonical Wnt signaling *in vitro* and *in vivo*. Its RNA polymerase activity may amplify certain ncRNAs, including promoting the generation of siRNAs. Moreover, increased expression of telomerase sensitizes mitochondrial DNA to oxidative damage and stimulates apoptosis. Thus, a polymorphism in *Canq1* may, secondarily or in *trans*, increase expression of telomerase in pial vessels of Bc embryos and contribute to reduced collateral formation and increased pruning. It is also intriguing that Nsmce1 and Ino80e interact with telomerase (discussed above), and that TGFβ1 and VEGF/Notch signaling are known to have significant cross talk with Wnt/β-catenin signaling [46].

Previously annotated mouse QTL identified within *Canq1* (128.3–142.2, MGI Mouse Genome Browser, Build 37) are unrelated to the collateral phenotype: *Alcp12* (alcohol preference,B6xA/J); coronin (actin binding protein-1A, B6xBXSB), lupus susceptibility (BXSB); *Myo1* (myocardial infarction susceptibility, BXSBxNZW, 142.2-extreme 3′ of *Canq1*); *Mob1*, (multigenic obesity, SpretxBc), *Rthyd1* (resistant to thymic deletion-1); *Scc12* (susceptibility to colon cancer 12); *Sluc19* (susceptibility to lung cancer-19); *Vpantd* (valproic acid-induced neural tube defect); *Dice2* (determination of IL4 commitment-2, B6xBc); *Il4ppq* (IL4 producing potential QTL, B6xBXSB, NZBxNZW, presumed same as *Dice2*); *Tsiq1* (T cell secretion of IL4-1m, presumed same as *Dice2*). Regarding the latter 3 QTL, Bc mice have ∼50-fold increased levels of IL4 compared to other strains, associated with a loss-of-function mutation which may explain their reduced mRNA (Fig. 6; Table S4) if resulting from negative feedback. No QTL or eQTL have been identified within the human genome syntenic to the *Canq1* interval, including in the 8 Mb flanking region (16p12.3-11.2/10q25-26.3) for the following traits: collateral circulation, stroke, ischemic stroke, cerebral circulation, coronary artery disease, angina, coronary ischemia, coronary circulation, coronary blood flow, myocardial infarction, peripheral artery disease, intermittent claudication (www.ncbi.nlm.nih.gov/omim; www.genome.gov/gwastudies/). The UCSC database identifies mir-762 at 134.85 and mir-1962 at the extreme 3′ position (142.8 Mb) of *Canq1*. In-situ hybridization of mir-762 was insufficient to permit structural assignment in the E14.5 mouse transcriptone atlas (http://www.eurexpress.org). The http://www.mirbase.org/ database lists only 3 deep-sequencing reads for miR-762 (1 in testes, 2 in brain). Since a miRNA of vascular importance would be expected to be reasonably expressed in most if not all tissues, these reports of very low expression in highly restricted tissue cDNAs, compared to other miRNAs, argue against the importance of mir-762 in Canq1.

Conclusions based on analysis of knockout mice and gene expression have limitations, including compensations from global deletion, difficulty in distinguishing gain-of-function variants, and altered expression dependent on changes elsewhere in the genome. In this regard, expression of *Flk1* and *Clic4* were not different in Bc and B6 embryos (Tables S3, S4, S5) despite their involvement in collateral formation based on targeted disruption [12], [17], [18].

In summary, the present results significantly narrow and prioritize the large number of candidate genes previously reported for the co-localized QTL *Canq1* [15] *Civq1* [16] and *HSq1* [19]. Additional studies sequencing EMMA-B and the wider *Canq1* interval in several strains with high and low collateral extent, generation of congenic strains partitioning EMMA and *Canq1*, and other approaches to investigate the above candidate genes, will be needed to identify the basis for this important locus for genetic-dependent variation in the collateral circulation.

## Supporting Information

Figure S1 **Pial collateral number per hemisphere and average diameter for 21 inbred strains, including 15 strains reported previously** [**9**] **.** Green bar denotes 6 newly phenotyped strains. Number of animals given at the base of each column.
(PDF)

Figure S2 **Heatmap of known and imputed SNPs within the EMMA region for 74 strains.** Abscissa, physical locations of selected SNPs across the EMMA region (p = 2.2×10^−5^). Ordinate, 74 inbred strains. 15 strains at bottom (bracket) arranged from least-to-most collateral number, per Zhang et al. [9]. Each vertical line represents a SNP (blue, SNP same as B6; green, different from B6; blank, genotype unknown).
(PDF)

Figure S3 **EMMA mapping using 20 strains excluding Cast/Ei.** Red and blue dots, the most significant and the second most significant SNPs in the previous EMMA mapping [15]. Same color scheme in Figures S3, S4, S5, S6, S7. Mapping allowed fewer than 3 SNPs with missing genotypes.
(PDF)

Figure S4 **EMMA mapping using 19 strains excluding Cast/Ei and PWD.** Mapping allowed fewer than 3 SNPs with missing genotypes.
(PDF)

Figure S5 **EMMA mapping using 18 strains excluding Cast/Ei, PWD, and LEWES.** Mapping allowed fewer than 3 SNPs with missing genotypes.
(PDF)

Figure S6 **EMMA mapping using 21 strains.** Mapping allowed no SNPs with missing genotypes.
(PDF)

Figure S7 **EMMA mapping using 21 strains with only informative SNPs.** Mapping allowed fewer than 3 SNPs with missing genotypes.
(PDF)

Figure S8 **IL-4 and Itgal knockout mice show no differences in perfusion, compared to their background strain, immediately after unilateral femoral artery ligation (FAL), indicating as in the pial circulation no effect on native collateral extent in skeletal muscle.** See Chalothorn and Faber [11] for Materials and Methods. IL-4, but not Itgal knockout mice, show deficits in recovery of perfusion and greater tissue ischemia and use-impairment with days after FAL. These deficiencies suggest impaired collateral remodeling, although lesser potential contributions could include less ischemic capillary angiogenesis and/or less reduction in resistance (smooth muscle tone or anatomic outward remodeling) upstream of, downstream of, or within the recruited hindlimb collateral network.
(PDF)

Table S1 **Primers for qRT-PCR.**
(PDF)

Table S2 **Differential blood count analysis in IL-4 and Itgal knockout mice, and C57BL/6 background strain.** Itgal knockouts show significant increases in cell counts for white blood cells, lymphocytes, granulocytes and monocytes, while platelet count is lower. *,**,*** p<0.05, 0.01, 0.001. Dunne et al and Ding et al reported similar increases in the above blood cell types in Itgal deficient mice, eg 3-fold for total leukocytes, 4-fold for PMNs, and 2.5-fold for mononuclear cells, but did not report platelets. Regarding the decrease in platelets, we have been unable to find any previous report that circulating platelet number is decreased in Itgal^−/−^ mice. However, Armugam et al reported that platelet adhesion to cerebral venules after MCA occlusion and reperfusion is 50% less in LFA−/− mice. Emoto et al found that platelets were reduced in livers following low dose LPS challenge in LFA−/− mice. Dunne JL, Collins RG, Beaudet AL, Ballantyne CM, Ley K. Mac-1, but not LFA-1, uses intercellular adhesion molecule-1 to mediate slow leukocyte rolling in TNF-alpha-induced inflammation. J Immunol. 2003;171:6105–11. Ding ZM, Ballantyne CM et al. Relative Contribution of LFA-1 and Mac-1 to Neutrophil Adhesion and Migration. J Immunol, 1999;163:5029–5038. Arumugam TV, Granger DN, et al. Contributions of LFA-1 and Mac-1 to brain injury and microvascular dysfunction induced by transient middle cerebral artery occlusion. AJP-Heart & Lung, 2004;287: H2555–H2560. Emoto M, Kaufmann SHE et al. Increased Resistance of LFA-1-Deficient Mice to Lipopolysaccharide-Induced Shock/Liver Injury in the Presence of TNF-α and IL-12 Is Mediated by IL-10: A Novel Role for LFA-1 in the Regulation of the Proinflammatory and Anti-Inflammatory Cytokine Balance. J Immunol. 2003;171:584–593.
(PDF)

Table S3 **ANOVA analysis of expression for 150 genes.** Expression assay by NanoString nCounter. 3 RNA samples of pia at each time point for each strain, with each sample composing of ≥8 embryos from ≥2 litters (18 samples, ∼200 embryos). Transcript number for each gene normalized to mean transcript number for 6 housekeeping genes: *βactin, Gapdh, Tubb5, Hprt1, Ppia, Tbp*. * Gene number same as in Figure 6 and Table 1. **^†^I**, genes in EMMA region; **II**, genes in 95% CI of Chr 7 QTL; **III**, angiogenic-related genes located elsewhere in genome; **IV**, proliferation-related genes located elsewhere in genome. **^‡^**Fold change for Bc vs B6 if positive and B6 vs Bc if negative. ^§^Bonferroni-adjusted p values (p-value÷150, for 150 genes/splice forms assayed) from 2-way ANOVA for 2 strains (20 genes p<0.05) and 3 embryonic days (6 genes p<0.05).
(PDF)

Table S4 **Genes having differential expression between strains by t-test analysis.** Expression assay by NanoString nCounter. 3 RNA samples of pia at each time point for each strain, with each sample composing of ≥8 embryos from ≥2 litters (18 samples, ∼200 embryos). Transcript number for each gene normalized to mean transcript number for 6 housekeeping genes: *βactin, Gapdh, Tubb5, Hprt1, Ppia, Tbp*. Gene number same as in Figure 6 and Table 1. **^†^I**, genes within the EMMA region; **II**, gene s in 95% CI of Chr 7 QTL; **III**, angiogenesis-related genes located elsewhere in genome; **IV**, proliferation-related genes located elsewhere in genome. **^‡^**Fold change for BALB/c vs B6 if positive and B6 vs BALB/c if negative. ^§^Bonferroni-adjusted p-values (p-value÷ 50) from t-tests independent of embryonic days (1 gene <0.05). ^∥^p values from t tests independent of embryonic days, without Bonferroni adjustment (49 genes <0.05).
(PDF)

Table S5 **T test analysis of expression for 150 genes.** Expression assay by NanoString nCounter. 3 RNA samples of pia at each time point for each strain, with each sample composing of ≥8 embryos from ≥2 litters (18 samples, ∼200 embryos). Transcript number for each gene normalized to mean transcript number for 6 housekeeping genes: *βactin, Gapdh, Tubb5, Hprt1, Ppia, Tbp*. • Gene number same as in Figure 6 and Table 1. **^†^** **I**, genes within the EMMA region; **II**, gene s in 95% CI of Chr 7 QTL; **III**, angiogenesis-related genes located elsewhere in genome; **IV**, proliferation-related genes located elsewhere in genome. **^‡^** Fold change for BALB/c vs B6 if positive and B6 vs BALB/c if negative. ^§^ Bonferroni-adjusted p-values (p-value÷ 50) from t-tests independent of embryonic days (1 gene <0.05). ^∥^ p values from t tests independent of embryonic days, without Bonferroni adjustment (49 genes <0.05).
(PDF)

Materials and Methods S1 (PDF)

## References

[1] Faber JE, Dai X, Lucitti J, Deindl E, Schaper W (2011). Genetic and environmental mechanisms controlling formation and maintenance of the native collateral circulation.. Arteriogenesis – Molecular Regulation, Pathophysiology and Therapeutics.

[2] Brozici M, van der Zwan A, Hillen B (2003). Anatomy and functionality of leptomeningeal anastomoses: A review.. Stroke.

[3] Shuaib A, Butcher K, Mohammad AA, Saqqur M, Liebeskind DS (2011). Collateral blood vessels in acute ischaemic stroke: a potential therapeutic target.. Lancet Neurol.

[4] de Marchi SF, Gloekler S, Meier P, Traupe T, Steck H (2011). Determinants of preformed collateral vessels in the human heart without coronary artery disease.. Cardiology.

[5] Schaper W (2009). Collateral circulation: Past and present.. Basic Research in Cardiology.

[6] Meier P, Gloekler S, Zbinden R, Beckh S, de Marchi SF (2007). Beneficial effect of recruitable collaterals: A 10-year follow-up study in patients with stable coronary artery disease undergoing quantitative collateral measurements.. Circulation.

[7] Menon BK, Smith EE, Modi J, Patel SK, Bhatia R (2011). Regional leptomeningeal score on CT angiography predicts clinical and imaging outcomes in patients with acute anterior circulation occlusions.. Am J Neuroradiol.

[8] Chalothorn D, Clayton JA, Zhang H, Pomp D, Faber JE (2007). Collateral density, remodeling, and vegf-a expression differ widely between mouse strains.. Physiol Genomics.

[9] Zhang H, Prabhakar P, Sealock R, Faber JE (2010). Wide genetic variation in the native pial collateral circulation is a major determinant of variation in severity of stroke.. J Cereb Blood Flow Metab.

[10] Chalothorn D, Faber JE (2010). Strain-dependent variation in collateral circulatory function in mouse hindlimb.. Physiol Genomics.

[11] Chalothorn D, Faber JE (2010). Formation and maturation of the native cerebral collateral circulation.. J Mol Cell Cardiol.

[12] Lucitti JL, Mackey J, Morrison J, Haigh J, Adams R (2012). Formation of the collateral circulation is regulated by vascular endothelial growth factor-A and a disintegrin and metalloprotease family members 10 and 17.. Circ Res.

[13] Dai X, Faber JE (2010). Endothelial nitric oxide synthase deficiency causes collateral vessel rarefaction and impairs activation of a cell cycle gene network during arteriogenesis.. Circ Res.

[14] Faber JE, Zhang H, Prabhakar, Lassance-Soares RM, Burnett MS (2011). Aging causes collateral rarefaction and increased severity of ischemic injury in multiple tissues.. Arterioscler Thromb Vasc Biol.

[15] Wang S, Zhang H, Dai X, Sealock R, Faber JE (2010). Genetic architecture underlying variation in extent and remodeling of the collateral circulation.. Circ Res.

[16] Keum S, Marchuk DA (2009). A locus mapping to mouse chromosome 7 determines infarct volume in a mouse model of ischemic stroke Circ.. Cardiovasc Genet.

[17] Clayton JA, Chalothorn D, Faber JE (2008). Vascular endothelial growth factor-A specifies formation of native collaterals and regulates collateral growth in ischemia.. Circ Res.

[18] Chalothorn D, Zhang H, Smith JE, Edwards JC, Faber JE (2009). Chloride intracellular channel-4 is a determinant of native collateral formation in skeletal muscle and brain.. Circ Res.

[19] Ayotunde OD, Keum S, Hazarika S, Youngun L, Lamonte GM (2008). A QTL (LSq-1) on mouse chromosome 7 is linked to the absence of tissue loss following surgical hind-limb ischemia.. Circulation.

[20] Kang HM, Zaitlen NA, Wade CM, Kirby A, Heckerman D (2008). Efficient control of population structure in model organism association mapping.. Genetics.

[21] Manichaikul A, Moon JY, Sen S, Yandell BS, Broman KW (2009). A model selection approach for the identification of quantitative trait loci in experimental crosses, allowing epistasis.. Genetics.

[22] Lynch M, Ritland K (1999). Estimation of pairwise relatedness with molecular markers.. Genetics.

[23] Hoover-Plow J, Shchurin A, Hart E, Sha J, Hill AE (2006). Genetic background determines response to hemostasis and thrombosis.. BMC Blood Disord.

[24] Geiss GK, Bumgarner RE, Birditt B, Dahl T, Dowidar N (2008). Direct multiplexed measurement of gene expression with color-coded probe pairs.. Nat Biotechnol.

[25] Malkov VA, Serikawa KA, Balantac N, Watters J, Geiss G (2009). Multiplexed measurements of gene signatures in different analytes using the nanostring ncounter assay system.. BMC Res Notes.

[26] Pfaffl MW, Horgan GW, Dempfle L (2002). Relative expression software tool (REST) for group-wise comparison and statistical analysis of relative expression results in real-time PCR.. Nucleic Acids Res.

[27] Shifman S, Bell JT, Copley RR, Taylor MS, Williams RW (2006). Mott R, Flint J. A high-resolution single nucleotide polymorphism genetic map of the mouse genome.. PLoS Biol.

[28] Faffe DS, Flynt L, Bourgeois K, Panettieri RA, Shore SA (2006). Interleukin-13 and interleukin-4 induce vascular endothelial growth factor release from airway smooth muscle cells: Role of vascular endothelial growth factor genotype.. Am J Respir Cell Mol Biol.

[29] Yamaji-Kegan K, Su Q, Angelini DJ, Johns RA (2009). Il-4 is proangiogenic in the lung under hypoxic conditions.. J Immunol.

[30] Paul WE, Zhu J (2010). How are t(h)2-type immune responses initiated and amplified?. Nat Rev Immunol.

[31] Mace EM, Monkley SJ, Critchley DR, Takei F (2009). A dual role for talin in NK cell cytotoxicity: Activation of lfa-1-mediated cell adhesion and polarization of NK cells.. J Immunol.

[32] Arumugam TV, Salter JW, Chidlow JH, Ballantyne CM, Kevil CG (2004). Contributions of lfa-1 and mac-1 to brain injury and microvascular dysfunction induced by transient middle cerebral artery occlusion.. Am J Physiol Heart Circ Physiol.

[33] Carmichael ST (2005). Rodent models of focal stroke: Size, mechanism, and purpose.. NeuroRx.

[34] Mohr JP (2004). Stroke: Pathophysiology, diagnosis, and management.

[35] Frazer KA, Eskin E, Kang HM, Bogue MA, Hinds DA (2007). A sequence-based variation map of 8.27 million snps in inbred mouse strains.. Nature.

[36] Yang H, Bell TA, Churchill GA, Pardo-Manuel de Villena F (2007). On the subspecific origin of the laboratory mouse.. Nature genetics.

[37] Kirby A, Kang HM, Wade CM, Cotsapas C, Kostem E (2010). Fine mapping in 94 inbred mouse strains using a high-density haplotype resource.. Genetics.

[38] Fujioka Y, Kimata Y, Nomaguchi K, Watanabe K, Kohno K (2002). Identification of a novel non-structural maintenance of chromosomes (SMC) component of the SMC5-SMC6 complex involved in DNA repair.. J Biol Chem.

[39] De Piccoli G, Torres-Rosell J, Aragón L (2009). The unnamed complex: what do we know about Smc5-Smc6?. Chromosome Res.

[40] Chavez A, George V, Agrawal V, Johnson FB (2010). Sumoylation and the structural maintenance of chromosomes (Smc) 5/6 complex slow senescence through recombination intermediate resolution.. J Biol Chem.

[41] Liu W, Tanasa B, Tyurina OV, Zhou TY, Gassmann R (2010). PHF8 mediates histone H4 lysine 20 demethylation events involved in cell cycle progression.. Nature.

[42] Noël JF, Wellinger RJ (2011). Abrupt telomere losses and reduced end-resection can explain accelerated senescence of Smc5/6 mutants lacking telomerase.. DNA Repair (Amst).

[43] Wardle FC, Papaioannou VE (2008). Teasing out T-box targets in early mesoderm.. Curr Opin Genet Dev.

[44] Toyo-oka K, Mori D, Yano Y, Shiota M, Iwao H (2008). Protein phosphatase 4 catalytic subunit regulates Cdk1 activity and microtubule organization via NDEL1 dephosphorylation.. J Cell Biol.

[45] Ghogomu SM, van Venrooy S, Ritthaler M, Wedlich D, Gradl D (2006). HIC-5 is a novel repressor of lymphoid enhancer factor/T-cell factor-driven transcription.. J Biol Chem.

[46] Dejana E (2010). The role of wnt signaling in physiological and pathological angiogenesis.. Circ Res.

[47] Chuai M, Weijer CJ (2009). Regulation of cell migration during chick gastrulation.. Curr Opin Genet Dev.

[48] Stalmans I, Lambrechts D, De Smet F, Jansen S, Wang J (2003). VEGF: a modifier of the del22q11 (DiGeorge) syndrome?. Nat Med.

[49] Wentzel P, Eriksson UJ (2009). Altered gene expression in neural crest cells exposed to ethanol in vitro.. Brain Res.

[50] Chen L, Cai Y, Jin J, Florens L, Swanson SK (2011). Subunit organization of the human INO80 chromatin remodeling complex: an evolutionarily conserved core complex catalyzes ATP-dependent nucleosome remodeling.. J Biol Chem.

[51] Morrison AJ, Shen X (2009). Chromatin remodelling beyond transcription: the INO80 and SWR1 complexes.. Nat Rev Mol Cell Biol.

[52] Davis BK, Wen H, Ting JP (2011). The inflammasome NLRs in immunity, inflammation, and associated diseases.. Annu Rev Immunol.

[53] Franchi L, Muñoz-Planillo R, Reimer T, Eigenbrod T, Núñez G (2010). Inflammasomes as microbial sensors.. Eur J Immunol.

[54] McElvania Tekippe E, Allen IC, Hulseberg PD, Sullivan JT (2010). Granuloma formation and host defense in chronic Mycobacterium tuberculosis infection requires PYCARD/ASC but not NLRP3 or caspase-1.. PLoS One.

[55] Numata K, Kanai A, Saito R, Kondo S, Adachi J (2003). Identification of putative noncoding RNAs among the RIKEN mouse full-length cDNA collection.. Genome Res.

[56] Loewer S, Cabili MN, Guttman M, Loh YH, Thomas K (2010). Large intergenic non-coding RNA-RoR modulates reprogramming of human induced pluripotent stem cells.. Nat Genet.

[57] Hung T, Wang Y, Lin MF, Koegel AK, Kotake Y (2011). Extensive and coordinated transcription of noncoding RNAs within cell-cycle promoters.. Nat Genet.

[58] Klose RJ, Kallin EM, Zhang Y (2006). JmjC-domain-containing proteins and histone demethylation.. Nat Rev Genet.

[59] Hsia DA, Tepper CG, Pochampalli MR, Hsia EY, Izumiya C (2010). KDM8, a H3K36me2 histone demethylase that acts in the cyclin A1 coding region to regulate cancer cell proliferation.. Proc Natl Acad Sci USA.

[60] Shi Y (2007). Histone lysine demethylases: emerging roles in development, physiology and disease.. Nat Rev Genet.

[61] de Lange T (2009). How telomeres solve the end-protection problem.. Science.

[62] Martínez P, Blasco MA (2011). Telomeric and extra-telomeric roles for telomerase and the telomere-binding proteins.. Nat Rev Cancer.

