# Supplementary material for: Genetic Dissection of the Canq1 Locus Governing Variation in Extent of the Collateral Circulation
Source: PLoS One. 2012 Mar 6;7(3):e31910. doi: 10.1371/journal.pone.0031910 (PMC3295810; doi:10.1371/journal.pone.0031910)

Figure S1. Pial collateral number per hemisphere and average diameter for 21 inbred strains, including 15 strains reported previously [9]. Green bar denotes 6 newly phenotyped strains. Number of animals given at the base of each column.

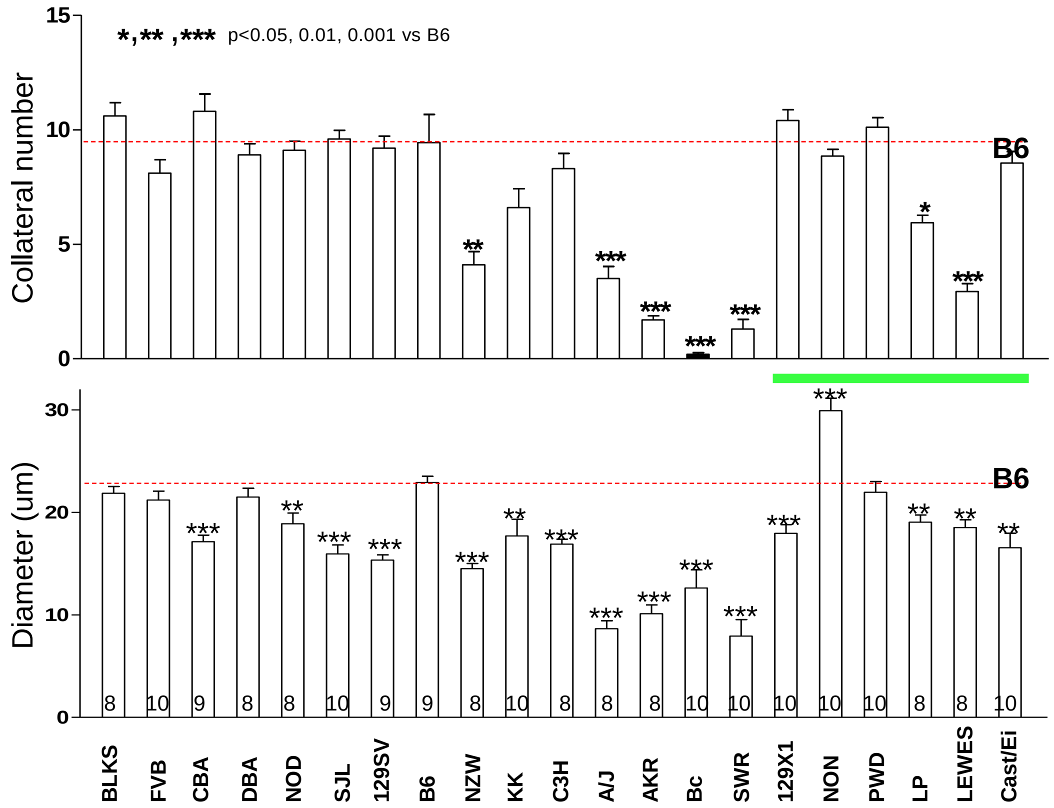

Supplement: Figure S1 — Pial collateral number per hemisphere and average diameter for 21 inbred strains, including 15 strains reported previously [9] . Green bar denotes 6 newly phenotyped strains. Number of animals given at the base of each column. (PDF) [file pone.0031910.s001.pdf]
