# Supplementary material for: Genetic Dissection of the Canq1 Locus Governing Variation in Extent of the Collateral Circulation
Source: PLoS One. 2012 Mar 6;7(3):e31910. doi: 10.1371/journal.pone.0031910 (PMC3295810; doi:10.1371/journal.pone.0031910)

Figure S3. EMMA mapping using 20 strains excluding Cast/Ei. Red and blue dots, the most significant and the second most significant SNPs in the previous EMMA mapping [15]. Same color scheme in Figures S3-S7. Mapping allowed fewer than 3 SNPs with missing genotypes.

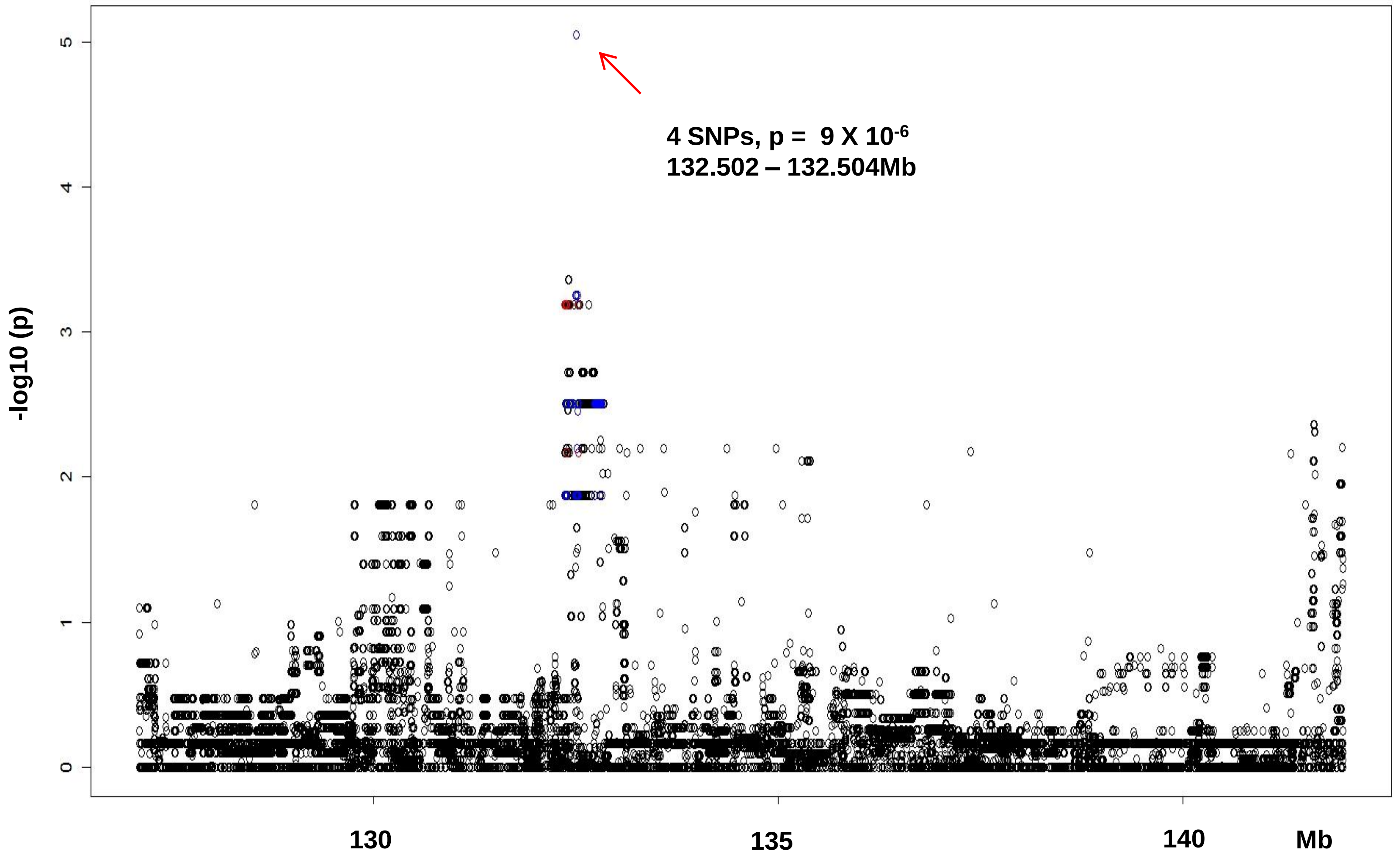

Supplement: Figure S3 — EMMA mapping using 20 strains excluding Cast/Ei. Red and blue dots, the most significant and the second most significant SNPs in the previous EMMA mapping [15]. Same color scheme in Figures S3, S4, S5, S6, S7. Mapping allowed fewer than 3 SNPs with missing genotypes. (PDF) [file pone.0031910.s003.pdf]
