# Supplementary material for: Genetic Dissection of the Canq1 Locus Governing Variation in Extent of the Collateral Circulation
Source: PLoS One. 2012 Mar 6;7(3):e31910. doi: 10.1371/journal.pone.0031910 (PMC3295810; doi:10.1371/journal.pone.0031910)

Figure S4. EMMA mapping using 19 strains excluding Cast/Ei and PWD. Mapping allowed fewer than 3 SNPs with missing genotypes.

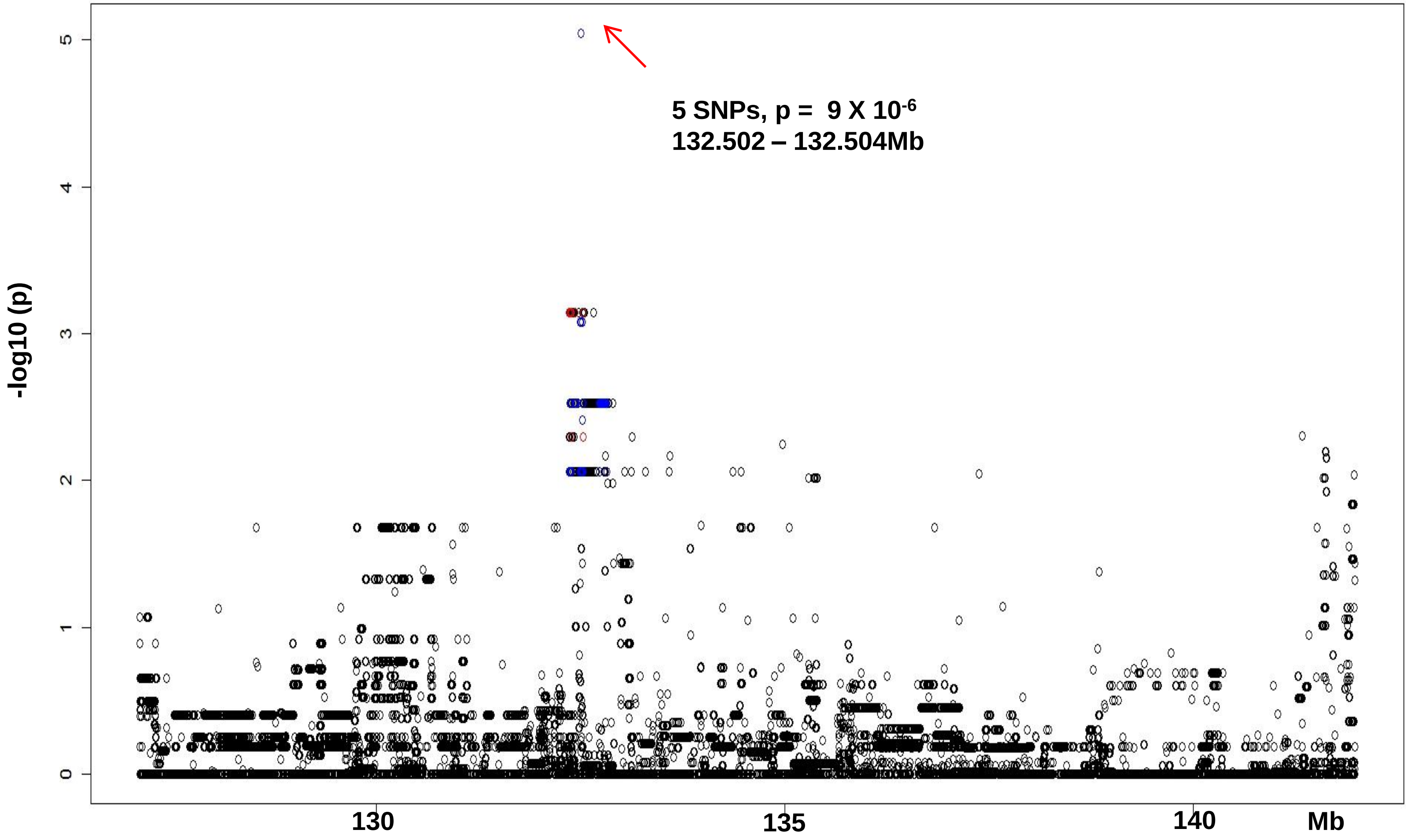

Supplement: Figure S4 — EMMA mapping using 19 strains excluding Cast/Ei and PWD. Mapping allowed fewer than 3 SNPs with missing genotypes. (PDF) [file pone.0031910.s004.pdf]
