# Supplementary figures and images for: Genetic Dissection of the Canq1 Locus Governing Variation in Extent of the Collateral Circulation
Source: PLoS One. 2012 Mar 6;7(3):e31910. doi: 10.1371/journal.pone.0031910 (PMC3295810; doi:10.1371/journal.pone.0031910)

Figure S6. EMMA mapping using 21 strains. Mapping allowed no SNPs with missing genotypes.

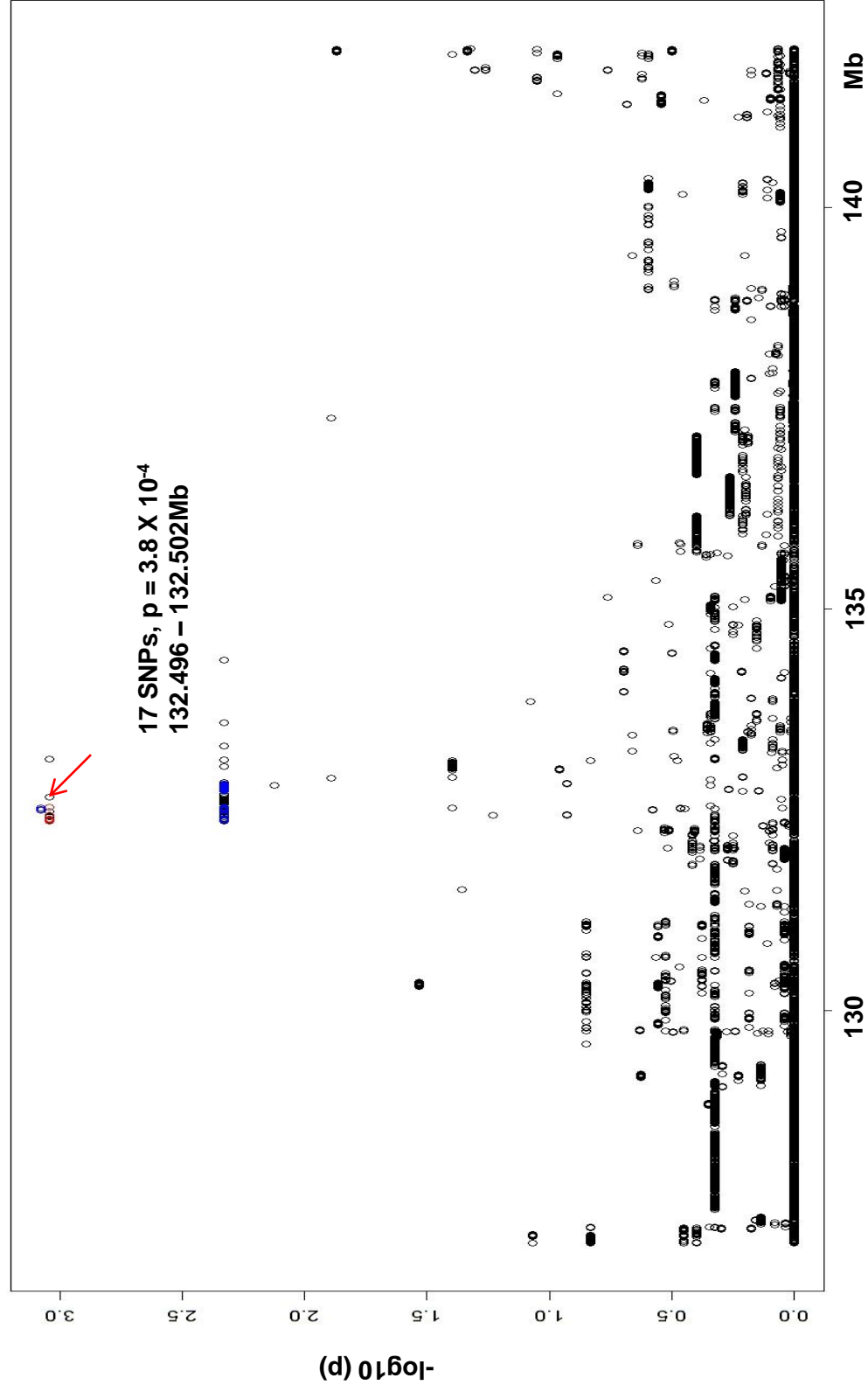

Supplement: Figure S6 — EMMA mapping using 21 strains. Mapping allowed no SNPs with missing genotypes. (PDF) [file pone.0031910.s006.pdf]
