# Supplementary material for: Genetic Dissection of the Canq1 Locus Governing Variation in Extent of the Collateral Circulation
Source: PLoS One. 2012 Mar 6;7(3):e31910. doi: 10.1371/journal.pone.0031910 (PMC3295810; doi:10.1371/journal.pone.0031910)
Supplement: Figure S8 — IL-4 and Itgal knockout mice show no differences in perfusion, compared to their background strain, immediately after unilateral femoral artery ligation (FAL), indicating as in the pial circulation no effect on native collateral extent in skeletal muscle. See Chalothorn and Faber [11] for Materials and Methods. IL-4, but not Itgal knockout mice, show deficits in recovery of perfusion and greater tissue ischemia and use-impairment with days after FAL. These deficiencies suggest impaired collateral remodeling, although lesser potential contributions could include less ischemic capillary angiogenesis and/or less reduction in resistance (smooth muscle tone or anatomic outward remodeling) upstream of, downstream of, or within the recruited hindlimb collateral network. (PDF) [file pone.0031910.s008.pdf]

Figure S8. IL-4 and Itgal knockout mice show no differences in perfusion, compared to their background strain, immediately after unilateral femoral artery ligation (FAL), indicating as in the pial circulation no effect on native collateral extent in skeletal muscle. See Chalothorn and Faber [11] for Materials and Methods. IL-4, but not Itgal knockout mice, show deficits in recovery of perfusion and greater tissue ischemia and use-impairment with days after FAL. These deficiencies suggest impaired collateral remodeling, although lesser potential contributions could include less ischemic capillary angiogenesis and/or less reduction in resistance (smooth muscle tone or anatomic outward remodeling) upstream of, downstream of, or within the recruited hindlimb collateral network.

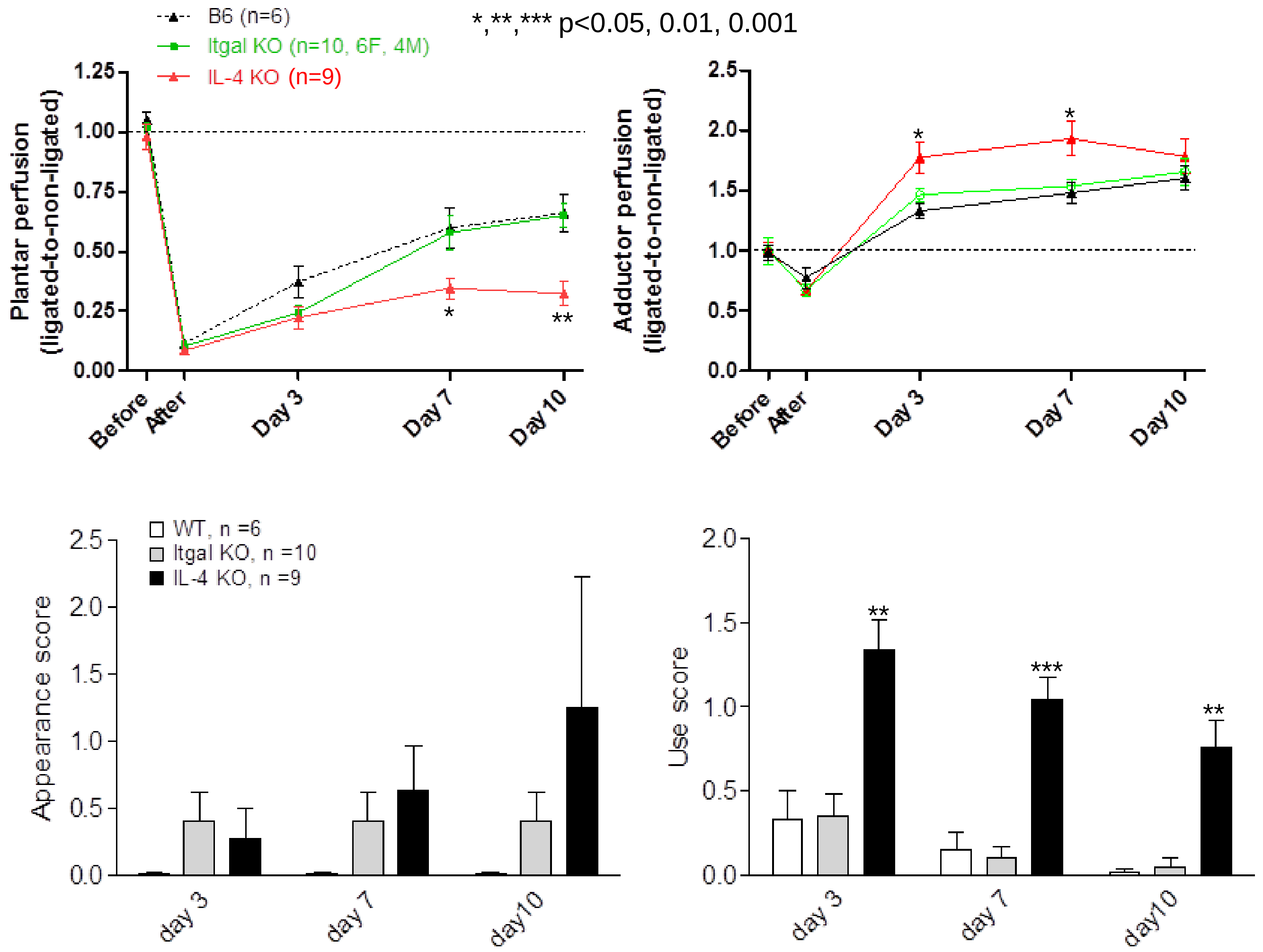

IL4$^{-/-}$ mice showed reduced recovery after femoral artery ligation, suggesting a role in collateral remodeling and possibly in ischemic angiogenesis. To our knowledge this is the first such report. Liu et al reported greater recovery from hindlimb ischemia in mice treated with bone marrow cells in association with higher serum levels of IL4 (see also Farivar et al 2003; Hellingman et al 2010).

Liu Q, Chen Z, Terry T, McNatt JM, Willerson JT, Zoldhelyi P. Intra-arterial transplantation of adult bone marrow cells restores blood flow and regenerates skeletal muscle in ischemic limbs. Vasc Endovascular Surg. 2009;43:433-43.
Farivar AS, Krishnadasan B, Naidu BV, Woolley SM, Verrier ED, Mulligan MS. Endogenous interleukin-4 and interleukin-10 regulate experimental lung ischemia reperfusion injury. Ann Thorac Surg. 2003;76:253-9.
Hellingman AA, Zwaginga JJ, van Beem RT; TeRM/Smart Mix Consortium, Hamming JF, Fibbe WE, Quax PH, Geutskens SB.T-cell-pre-stimulated monocytes promote neovascularisation in a murine hind limb ischaemia model. Eur J Vasc Endovasc Surg. 2011;41:418-28.
