# Supplementary material for: Genetic Dissection of the Canq1 Locus Governing Variation in Extent of the Collateral Circulation
Source: PLoS One. 2012 Mar 6;7(3):e31910. doi: 10.1371/journal.pone.0031910 (PMC3295810; doi:10.1371/journal.pone.0031910)
Supplement: Table S1 — Primers for qRT-PCR. (PDF) [file pone.0031910.s009.pdf]

Online table I. Primers for qRT-PCR

Gene	Sense	Antisense
Angpt2	*5'*-AGC AGA TTT TGG ATC ACA CCAG	5'-GCT CCT TCA TGG ACT GTA GCTG-3'
Klf4	5'-TGC CAG ACC AGA TGC AGT CAC-3',	5'-GTA GTG CCT GGT CAG TTC ATC- 3'
Tgfb1	5′-GCT ACC ATG CCA ACT TCT GTCT-3′	5′-CCT ACC ACC CCA GCC TCTG-3′
