# Supplementary material for: Genetic Dissection of the Canq1 Locus Governing Variation in Extent of the Collateral Circulation
Source: PLoS One. 2012 Mar 6;7(3):e31910. doi: 10.1371/journal.pone.0031910 (PMC3295810; doi:10.1371/journal.pone.0031910)
Supplement: Table S2 — Differential blood count analysis in IL-4 and Itgal knockout mice, and C57BL/6 background strain. Itgal knockouts show significant increases in cell counts for white blood cells, lymphocytes, granulocytes and monocytes, while platelet count is lower. *,**,*** p<0.05, 0.01, 0.001. Dunne et al and Ding et al reported similar increases in the above blood cell types in Itgal deficient mice, eg 3-fold for total leukocytes, 4-fold for PMNs, and 2.5-fold for mononuclear cells, but did not report platelets. Regarding the decrease in platelets, we have been unable to find any previous report that circulating platelet number is decreased in Itgal−/− mice. However, Armugam et al reported that platelet adhesion to cerebral venules after MCA occlusion and reperfusion is 50% less in LFA−/− mice. Emoto et al found that platelets were reduced in livers following low dose LPS challenge in LFA−/− mice. Dunne JL, Collins RG, Beaudet AL, Ballantyne CM, Ley K. Mac-1, but not LFA-1, uses intercellular adhesion molecule-1 to mediate slow leukocyte rolling in TNF-alpha-induced inflammation. J Immunol. 2003;171:6105–11. Ding ZM, Ballantyne CM et al. Relative Contribution of LFA-1 and Mac-1 to Neutrophil Adhesion and Migration. J Immunol, 1999;163:5029–5038. Arumugam TV, Granger DN, et al. Contributions of LFA-1 and Mac-1 to brain injury and microvascular dysfunction induced by transient middle cerebral artery occlusion. AJP-Heart & Lung, 2004;287: H2555–H2560. Emoto M, Kaufmann SHE et al. Increased Resistance of LFA-1-Deficient Mice to Lipopolysaccharide-Induced Shock/Liver Injury in the Presence of TNF-α and IL-12 Is Mediated by IL-10: A Novel Role for LFA-1 in the Regulation of the Proinflammatory and Anti-Inflammatory Cytokine Balance. J Immunol. 2003;171:584–593. (PDF) [file pone.0031910.s010.pdf]

Table S2. Differential blood count analysis in IL-4 and Itgal knockout mice, and C57BL/6 background strain. Itgal knockouts show significant increases in cell counts for white blood cells, lymphocytes, granulocytes and monocytes, while platelet count is lower. *,**,*** p<0.05, 0.01, 0.001

		WBC 10^3/ul	LYMF 10^3/ul	GRAN 10^3/ul	MONO 10^3/ul	LYMF %	GRAN %	MONO %	HCT %
C57	Aveg	5.12	4.18	0.53	0.52	79.35	16.62	13.06	37.19
n=11	SE	0.49	0.45	0.04	0.07	2.82	4.64	2.37	1.33
IL-4 KO	Aveg	4.85	3.92	0.45	0.58	79.45	11.00	11.46	31.95
n=6	SE	1.05	0.94	0.10	0.10	5.38	2.98	1.91	1.34
Itgal KO	Aveg	*** 11.62	*** 8.62	** 1.52	** 1.48	75.08	12.90	12.02	36.20
n=10	SE	1.20	0.80	0.30	0.26	2.89	1.67	1.53	0.68

		MCV fl	RBC 10^6/ul	HGB g/dl	MCH pg	MCHC g/dl	RDW %	MPV fl	PLT 10^3/ul
C57	Aveg	38.44	9.48	14.15	15.57	38.32	18.84	6.59	530.07
n=11	SE	2.22	0.50	0.44	0.21	0.54	0.24	0.07	45.80
IL-4 KO	Aveg	40.93	7.81	12.93	16.62	40.65	20.18	6.67	658.67
n=6	SE	0.17	0.35	0.52	0.49	1.18	0.68	0.10	46.96
Itgal KO	Aveg	41.04	8.82	14.35	16.31	39.76	18.94	6.79	* 384.20
n=10	SE	0.22	0.16	0.18	0.23	0.51	0.35	0.09	51.22

Dunne et al and Ding et al reported similar increases in the above blood cell types in Itgal deficient mice, eg 3-fold for total leukocytes, 4-fold for PMNs, and 2.5-fold for mononuclear cells, but did not report platelets. Regarding the decrease in platelets, we have been unable to find any previous report that circulating platelet number is decreased in $Itgal^{-/-}$ mice. However, Armugam et al reported that platelet adhesion to cerebral venules after MCA occlusion and reperfusion is 50% less in LFA-/- mice. Emoto et al found that platelets were reduced in livers following low dose LPS challenge in LFA-/- mice.

Dunne JL, Collins RG, Beaudet AL, Ballantyne CM, Ley K. Mac-1, but not LFA-1, uses intercellular adhesion molecule-1 to mediate slow leukocyte rolling in TNF-alpha-induced inflammation. J Immunol. 2003 ;171:6105-11.

Ding ZM, Ballantyne CM et al. Relative Contribution of LFA-1 and Mac-1 to Neutrophil Adhesion and Migration. J Immunol, 1999;163:5029-5038.

Arumugam TV, Granger DN, et al. Contributions of LFA-1 and Mac-1 to brain injury and microvascular dysfunction induced by transient middle cerebral artery occlusion. AJP-Heart & Lung, 2004;287: H2555-H2560.

Emoto M, Kaufmann SHE et al. Increased Resistance of LFA-1-Deficient Mice to Lipopolysaccharide-Induced Shock/Liver Injury in the Presence of TNF-α and IL-12 Is Mediated by IL-10: A Novel Role for LFA-1 in the Regulation of the Proinflammatory and Anti-Inflammatory Cytokine Balance. J Immunol. 2003;171:584-593.
