# Supplementary material for: Genetic Dissection of the Canq1 Locus Governing Variation in Extent of the Collateral Circulation
Source: PLoS One. 2012 Mar 6;7(3):e31910. doi: 10.1371/journal.pone.0031910 (PMC3295810; doi:10.1371/journal.pone.0031910)
Supplement: Table S3 — ANOVA analysis of expression for 150 genes. Expression assay by NanoString nCounter. 3 RNA samples of pia at each time point for each strain, with each sample composing of ≥8 embryos from ≥2 litters (18 samples, ∼200 embryos). Transcript number for each gene normalized to mean transcript number for 6 housekeeping genes: βactin, Gapdh, Tubb5, Hprt1, Ppia, Tbp. * Gene number same as in Figure 6 and Table 1. †I, genes in EMMA region; II, genes in 95% CI of Chr 7 QTL; III, angiogenic-related genes located elsewhere in genome; IV, proliferation-related genes located elsewhere in genome. ‡Fold change for Bc vs B6 if positive and B6 vs Bc if negative. §Bonferroni-adjusted p values (p-value÷150, for 150 genes/splice forms assayed) from 2-way ANOVA for 2 strains (20 genes p<0.05) and 3 embryonic days (6 genes p<0.05). (PDF) [file pone.0031910.s011.pdf]

Table S3. ANOVA analysis of expression for 150 genes

Gene* numbe	Name	Accession number	Gene[†] Group	Start	Fold[‡] E14.5	Fold[‡] E16.5	Fold[‡] E18.5	Bonadj[§] strain	Bonadj[§] Eday
1	EG545999	XM_620562.3	I	132218849	-1.02	1.3	1.33	1	1
2	LOC670828	LOC670828.1	I	132362963	1.44	1.12	1.84	1	1
3	4930533L02Rik	AK015957.1	I	132461873	-1.33	1.13	1.41	1	1
4	4933440M02Rik	XR_035453.1	I	132473157	1.48	-1.16	1.25	1	1
5	Jmjd5-001	ENSMUST00000033010.1	I	132588190	1.19	1.35	1.3	1	1
6	Jmjd5-002	ENSMUST00000135129.1	I	132588190	-1.62	1.07	1.31	1	1
7	Nsmce1-001	ENSMUST00000033006.1	I	132611154	1.36	1.36	1.66	0.0852	1
8	Nsmce1-002	ENSMUST00000138616.1	I	132611154	2.52	2.05	3.57	0.0122	1
9	Nsmce1-003	ENSMUST00000149289.1	I	132611154	5.54	3.58	7.09	0.0084	1
10	EG244214	XR_002216.2	I	132690783	1.26	1.01	1.69	1	1
11	Il4ra-201	ENSMUST00000033004.1	I	132695785	-1.92	-1.93	-1.02	0.7669	1
12	Il21r-201	ENSMUST00000033000.1	I	132746983	-1.19	-1.1	-1.06	1	1
13	Il21r-202	ENSMUST00000084605.1	I	132746983	1.07	1.17	-1.19	1	1
14	Gtf3c1-201	ENSMUST00000055506.1	I	132784469	1.25	1.19	1.47	0.1134	0.1431
15	Gtf3c1-202	ENSMUST00000106423.1	I	132784469	1.04	-1.09	1.09	1	1
16	D430042O09Rik-204	ENSMUST00000122337.1	II	132851390	3.94	2.61	3.65	0.0073	1
17	D430042O09Rik-205	ENSMUST00000124223.1	II	132851390	1.13	-1.06	-1.14	1	1
18	D430042O09Rik-206	ENSMUST00000132204.1	II	132851390	1.24	-1.04	1.11	1	1
19	D430042O09Rik-209	ENSMUST00000155059.1	II	132851390	2.04	2.09	5.63	0.0284	1
20	AC150648.2	NM_001101488.1	II	133024217	-1.59	-1.28	-1.27	1	0.0448

21	Xpo6	NM_028816.2	II	133245237	1.49	1.25	1.63	0.0861	1
22	Sbk1	NM_145587.2	II	133416133	1.08	-1.11	-1.09	1	1
23	AC135809.1	XM_001479519.1	II	133442948	1.09	1.11	1.55	1	1
24	Lat	NM_010689.2	II	133507346	-1.34	1.34	1.09	1	1
25	Spns1	NM_023712.2	II	133513574	-1.25	1.11	1.23	1	1
26	Nfatc2ip	NM_010900.2	II	133526368	1.68	1.69	2.05	0.0125	1
27	Cd19	NM_009844.2	II	133552519	1.38	1.63	1.51	1	0.9776
28	Rabep2	NM_030566.2	II	133572273	1.31	1.41	2.22	0.2858	1
29	Atp2a1	NM_007504.2	II	133589372	1.38	1.15	1.34	1	1
30	Sh2b1	NM_001081459.1	II	133610508	1.18	1.09	1.36	1	1
31	Tufm	NM_172745.3	II	133630892	1.18	1.17	1.61	1	1
32	Atxn2l	NM_183020.1	II	133635224	1.46	1.21	1.52	0.1097	1
33	Eif3c	NM_146200.1	II	133690426	1.34	1.3	1.63	0.1887	1
34	Cln3	NM_001146311.1	II	133714721	1.6	1.28	2.23	0.0697	1
35	Apob48r	NM_138310.1	II	133728456	-1.07	1.12	1.38	1	1
36	Il27	NM_145636.1	II	133732524	1.44	-1.15	1.17	1	1
37	Nupr1	NM_019738.1	II	133766763	-1.72	1.21	1.7	1	0.0096
38	2510046G10Rik	ENSMUST00000084587.1	II	133792737	1.26	1.26	1.75	0.6995	1
39	Ccdc101	NM_029339.2	II	133792823	1.5	1.5	2.21	0.2086	1
40	Sult1a1	NM_133670.1	II	133816379	-2.29	1.01	1.76	1	0.0026

41	Giyd2	NM_029420.3	II	133832982	1.57	1.63	2.4	0.0291	1
42	Bola2	NM_175103.3	II	133838915	1.35	1.26	1.75	0.4726	1
43	Coro1a	NM_009898.2	II	133843287	1.15	1.05	1.42	1	0.4048
44	Mapk3	NM_011952.2	II	133903115	1.05	1.18	1.41	1	1
45	Gdpd3	NM_024228.2	II	133909928	1.74	1.35	2	0.8226	1
46	Ypel3	NM_026875.2	II	133920469	1.19	1.15	1.66	0.3037	1
47	Tbx6	NM_011538.2	II	133924997	2.38	2.09	2.89	0.0055	1
48	Ppp4c	NM_019674.3	II	133929421	1.16	1.18	1.33	1	1
49	Aldoa	NM_007438.3	II	133938748	1.25	1.04	1.58	1	1
50	Fam57b-201	ENSMUST00000079423.1	II	133963344	1.79	-1.08	1.55	1	1
51	Fam57b-202	ENSMUST00000098032.1	II	133963344	1.23	1.36	1.63	1	1
52	4930451I11Rik	NM_183131.2	II	133973988	-1.22	-1.3	1.19	1	1
53	Doc2a	NM_010069.1	II	133990930	3.7	-1.27	1.94	1	1
54	Ino80e	NM_153580.1	II	133995094	4.64	2.71	3.79	0.0343	1
55	Taok2	NM_001163774.1	II	134009192	1.65	1.38	1.97	0.0143	1
56	Tmem219	NM_028389.1	II	134029685	1.48	1.39	1.99	0.1386	1
57	Kctd13	NM_172747.2	II	134072393	1.26	-1.02	1.28	1	1
58	Sez6l2	NM_144926.4	II	134085658	1.36	-1.23	1.24	1	1
59	Asphd1	NM_001039645.1	II	134089081	1.7	1.08	1.61	1	1
60	Cdipt	NM_138754.3	II	134119902	1.06	1.28	1.5	1	1

61	Mvp	NM_080638.2	II	134130382	-1.26	1.13	1.21	1	1
62	2900092E17Rik	NM_030240.1	II	134144996	1.71	1.43	2.42	0.1198	1
63	Kif22	NM_145588.1	II	134171246	1.37	1.45	1.71	1	1
64	AI467606	NM_178901.3	II	134234873	-1.01	1.35	1.62	1	1
65	Qprt-001	ENSMUST00000032912.1	II	134250628	-1.55	-1.19	1.1	1	0.0377
66	Qprt-002	ENSMUST00000129332.1	II	134250628	1.35	1.72	1.81	0.2412	0.1273
67	Qprt-003	ENSMUST00000142403.1	II	134250628	1.61	2.19	4.1	1	1
68	Cd2bp2	NM_027353.3	II	134337200	1.32	1.23	1.46	0.0554	1
69	Tbc1d10b	NM_144522.5	II	134340979	1.01	-1.02	1.18	1	1
70	Mylpf	NM_016754.5	II	134355122	1.17	1.14	1.62	1	1
71	Sept1	NM_017461.2	II	134357961	1.33	1.68	1.66	0.2131	1
72	AC133494.1	NR_030674.1	II	134376338	3.03	1.9	3.23	0.0625	1
73	Zfp553	NM_146201.1	II	134376575	1.02	1.05	1.06	1	0.8373
74	Zfp771	NM_177362.3	II	134388040	1.16	1.05	1.31	1	1
75	Dctpp1	NM_023203.1	II	134400473	1.38	1.11	1.48	1	1
76	Sephs2	NM_009266.3	II	134416075	1.14	1.12	1.27	1	1
77	Itgal-003	ENSMUST00000120857.1	II	134439774	1.82	2.09	1.76	1	1
78	Zfp768	NM_146202.1	II	134486312	1.22	-1.02	1.65	1	1
79	Zfp747	NM_175560.3	II	134516078	1.49	1.42	1.98	0.0991	1
80	9130019O22Rik	NM_030226.2	II	134525774	3.63	2.28	3.78	0.019	1

81	AC133494.2	NM_198011.1	II	134527268	1.35	1.15	1.65	0.8822	1
82	Zfp764	NM_146203.3	II	134547182	1.4	1.44	2.1	0.1708	1
83	Zfp689	NM_175163.3	II	134587266	1.31	1.44	1.9	0.2063	1
84	Prr14	NM_145589.2	II	134603125	1.26	1.12	1.64	0.7564	1
85	Fbrs	NM_010183.1	II	134629170	1.22	1.14	1.46	0.2593	1
86	Srcap	XM_001480403.1	II	134655516	1.33	1.15	1.53	0.255	1
87	1700008J07Rik	NR_024331.1	II	134655683	2.64	1.87	3.05	0.0202	1
88	Phkg2	NM_026888.3	II	134716854	1.7	1.34	2.19	0.025	1
89	Gm166	NM_001033040.2	II	134726602	2.31	1.63	2.26	0.2001	1
90	Rnf40	NM_172281.1	II	134732281	1.18	1.25	1.42	0.1315	1
91	1700120K04Rik	NR_027915.1	II	134747592	3.37	1.99	2.35	0.0177	1
92	Zfp629	NM_177226.5	II	134750545	1.24	1.08	1.62	1	1
93	Bcl7c	NM_009746.2	II	134806867	-1.05	-1.01	1	1	1
94	Ctf1	NM_007795.1	II	134856258	1.53	1.1	1.54	0.2593	0.1427
95	Ctf2	NM_198858.1	II	134861620	1.32	1.05	1.15	1	1
96	Fbxl19	NM_172748.2	II	134890289	1.47	1.07	1.5	1	0.8551
97	Orai3	NM_198424.3	II	134913329	2	1.59	2.73	0.1136	1
98	Setd1a	NM_178029.3	II	134920184	1.39	1.3	1.58	0.1413	1
99	Hsd3b7	NM_133943.2	II	134929122	1.06	1.27	1.53	1	0.2238
100	Stx1b	NM_024414.2	II	134947414	1.93	1.03	1.76	1	1

101	Stx4a	NM_009294.3	II	134967808	1.34	1.41	1.88	0.7125	1
102	Zfp668	NM_146259.3	II	135008684	1.7	1.21	1.82	0.1367	1
103	Zfp646	NM_172749.4	II	135020310	1.35	1.31	1.59	0.076	1
104	BC039632	NM_001081268.1	II	135029355	1.88	1.29	2.15	1	1
105	Vkorc1	NM_178600.2	II	135029741	1.07	1.27	1.68	1	1
106	Myst1	NM_026370.1	II	135056031	1.56	1.33	2.1	0.0889	1
107	Prss8	NM_133351.2	II	135069232	-1.26	1.5	1.71	1	1
108	Prss36	NM_001081374.1	II	135076152	1.15	1.25	1.65	1	1
109	Fus	NM_139149.2	II	135110971	1.47	1.49	1.72	0.0428	0.6911
110	B230325K18Rik	NM_176936.2	II	135126593	9.69	4.68	7.75	0.0165	1
111	Pycard	NM_023258.4	II	135135617	-2.29	-1.67	-1.79	0.0003	0.0595
112	Trim72	NM_001079932.2	II	135147503	2.19	2.07	2.27	0.7767	1
113	Itgax	NM_021334.2	II	135273061	1.25	1.75	1.36	1	1
114	Itgad	NM_001029872.1	II	135298292	1.65	-1.07	1.06	1	1
115	Cox6a2	NM_009943.2	II	135349128	1.05	1.18	1.76	1	1
116	9130023H24Rik	NM_177001.3	II	135379920	1.21	1.39	1.74	0.6463	1
117	Armc5	NM_146205.2	II	135381630	-1.15	1.03	1.07	1	1
118	Tgfb1i1	NM_009365.2	II	135390385	5.01	4.35	5.85	0.0634	1
119	Slc5a2	NM_133254.3	II	135409171	1.14	1.29	1.5	1	1
120	BC017158	NM_145590.1	II	135414893	1.56	1.43	1.67	0.0226	1

121	Rgs10	NM_026418.2	II	135517135	1.1	-1.22	1.31	1	1
122	Tial1	NM_009383.2	II	135583291	2.02	1.54	2.12	0.0418	1
123	Bag3	NM_013863.4	II	135667130	-1.42	-1.16	-1.15	1	1
124	Inpp5f	NM_178641.5	II	135754842	1.9	1.69	2.3	0.0036	1
125	Ctbp2	NM_001170744.1	II	140179304	1.22	1.15	1.33	1	1
126	LOC100043248	XM_001479821.1	II	141616800	2.49	-1.04	1.31	1	1
127	Dock1	NM_001033420.2	II	141862370	1.87	2.15	2.47	0.0384	1
128	Angpt1	NM_009640.3	III	42256273	1.05	1.01	1.66	1	1
129	Angpt2	NM_007426.3	III	18690263	-1.15	1.14	1.61	1	0.0077
130	Clic4	NM_013885.2	III	134769884	-1.17	1.08	1.25	1	1
131	Dll4	NM_019454.2	III	119151520	1.09	1.43	1.6	1	1
132	Ephrinb2	NM_010111.5	III	8617434	1.24	1.02	1.05	1	1
133	Flk1	NM_010612.2	III	76328852	1.13	1.46	1.61	1	1
134	Flt1	NM_010228.3	III	148373180	1.15	1.86	2.15	0.2966	1
135	Klf2	NM_008452.2	III	74842932	-1.66	1.17	-1.31	1	0.8971
136	Klf4	NM_010637.3	III	55540015	-1.43	1.18	1.29	1	0.0591
137	Pdgfa	NM_008808.3	III	139451968	1.43	1.11	1.29	1	1
138	Pdgfb	NM_011057.3	III	79826330	-1.18	1.32	1.12	1	1
139	Tgfb1	NM_019919.2	III	75404869	1.3	1.18	1.32	0.1245	0.0087
140	Vegfa188	NM_001025250.3	III	46153942	1.88	1.45	2.05	0.0236	1

141	Ampk	NM_001013367.3	IV	5093861	1.35	1.3	1.69	0.4531	1
142	Ki67	NM_001081117.2	IV	142881772	1.37	1.65	1.55	0.9798	1
143	Lkb1	NM_011492.3	IV	79578548	1.15	1.11	1.26	1	1
144	p16INK4a	NM_009877.2	IV	88920377	1.44	1.39	1.3	1	1
145	p21	NM_007669.4	IV	29227924	1.33	1.13	1.32	1	1
146	p27	NM_009875.4	IV	134870419	1.4	1.22	1.68	0.0893	1
147	p53	NM_011640.1	IV	69393861	1.22	1.39	1.6	0.2812	1
148	Pcna	NM_011045.2	IV	132074898	1.31	1.11	1.33	1	1
149	Sirt1	NM_019812.1	IV	62781753	1.48	1.14	1.57	1	1
150	telomerase	NM_009354.1	IV	73764438	3.99	2.14	3.87	0.0085	1

Expression assay by NanoString nCounter. 3 RNA samples of pia at each time point for each strain, with each sample composing of ≥ 8 embryos from ≥ 2 litters (18 samples, ~200 embryos). Transcript number for each gene normalized to mean transcript number for 6 housekeeping genes: *βactin, Gapdh, Tubb5, Hprt1, Ppia, Tbp.*

***** Gene number same as in Figure 6 and Table 1.

† **I**, genes in EMMA region; **II**, genes in 95% CI of Chr 7 QTL; **III**, angiogenic-related genes located elsewhere in genome; **IV**, proliferation-related genes located elsewhere in genome

‡ Fold change for Bc vs B6 if positive and B6 vs Bc if negative.

§ Bonferroni-adjusted p values (p-value÷150, for 150 genes/splice forms assayed) from 2-way ANOVA for 2 strains (20 genes p<0.05) and 3 embryonic days (6 genes p<0.05).
