# Supplementary material for: Genetic Dissection of the Canq1 Locus Governing Variation in Extent of the Collateral Circulation
Source: PLoS One. 2012 Mar 6;7(3):e31910. doi: 10.1371/journal.pone.0031910 (PMC3295810; doi:10.1371/journal.pone.0031910)
Supplement: Table S4 — Genes having differential expression between strains by t-test analysis. Expression assay by NanoString nCounter. 3 RNA samples of pia at each time point for each strain, with each sample composing of ≥8 embryos from ≥2 litters (18 samples, ∼200 embryos). Transcript number for each gene normalized to mean transcript number for 6 housekeeping genes: βactin, Gapdh, Tubb5, Hprt1, Ppia, Tbp. Gene number same as in Figure 6 and Table 1. †I, genes within the EMMA region; II, gene s in 95% CI of Chr 7 QTL; III, angiogenesis-related genes located elsewhere in genome; IV, proliferation-related genes located elsewhere in genome. ‡Fold change for BALB/c vs B6 if positive and B6 vs BALB/c if negative. §Bonferroni-adjusted p-values (p-value÷ 50) from t-tests independent of embryonic days (1 gene <0.05). ∥p values from t tests independent of embryonic days, without Bonferroni adjustment (49 genes <0.05). (PDF) [file pone.0031910.s012.pdf]

Table S4. Genes having differential expression between strains by t-test analysis.

Gene* number	Name	Description	Gene† Group	Start	Fold‡	Bonadj§	P value‖
111	Pycard	PYD and CARD domain containing Gene	II	135135617	-2.24	0.006	0.00004
11	Il4ra-201	interleukin 4 receptor, alpha	I	132695785	-1.82	0.062	0.00041
91	1700120K04Rik	RIKEN cDNA 1700120K04 gene	II	134747592	2.11	0.104	0.00069
124	Inpp5f	inositol polyphosphate-5-phosphatase F	II	135754842	1.65	0.121	0.00081
47	Tbx6	T-box 6	II	133924997	2.06	0.150	0.00100
9	Nsmce1-003	non-SMC element 1 homolog	I	132611154	4.35	0.166	0.00111
123	Bag3	BCL2-associated athanogene 3	II	135667130	-1.38	0.200	0.00134
80	9130019O22Rik	RIKEN cDNA E430018J23 gene	II	134525774	2.63	0.248	0.00166
110	B230325K18Rik	RIKEN cDNA B230325K18	II	135126593	6.13	0.273	0.00182
8	Nsmce1-002	non-SMC element 1 homolog	I	132611154	2.21	0.327	0.00218
150	telomerase	telomerase reverse transcriptase	IV	73764438	2.87	0.340	0.00227
16	D430042O09Rik-204	RIKEN cDNA D430042O09 gene	II	132851390	2.87	0.349	0.00232
87	1700008J07Rik	non-coding RNA	II	134655683	2.10	0.395	0.00263
54	Ino80e	INO80 complex subunit E	II	133995094	3.01	0.474	0.00316
12	Il21r-201	interleukin 21 receptor, alpha	I	132746983	-1.29	0.485	0.00323
26	Nfatc2ip	nuclear factor calcineurin-dependent 2 interacting protein	II	133526368	1.52	0.489	0.00326
19	D430042O09Rik-209	RIKEN cDNA D430042O09 gene	II	132851390	2.43	0.539	0.00360
118	Tgfb1i1	transforming growth factor beta 1	II	135390385	4.19	0.549	0.00366
88	Phkg2	phosphorylase kinase, gamma 2	II	134716854	1.46	0.554	0.00369
55	Taok2	TAO kinase 2	II	134009192	1.42	0.569	0.00380

127	Dock1	dedicator of cytokinesis 1	II	141862370	1.77	0.615	0.00410
41	Giyd2	GIY-YIG domain containing 2	II	133832982	1.60	0.681	0.00454
34	Cln3	ceroid lipofuscinosis, neuronal 3	II	133714721	1.46	0.817	0.00545
72	AC133494.1	Gm4532 predicted gene	II	134376338	2.29	0.868	0.00579
122	Tial1	Tia1 cytotoxic granule-associated RNA binding protein-like 1	II	135583291	1.58	0.893	0.00595
140	Vegfa188	vascular endothelial growth factor A	III	46153942	1.51	1.000	0.00772
120	BC017158	UPF0420 protein C16orf58 homolog	II	135414893	1.33	1.000	0.00827
62	2900092E17Rik	RIKEN cDNA 2900092E17 gene	II	134144996	1.56	1.000	0.01063
112	Trim72	tripartite motif-containing 72	II	135147503	1.91	1.000	0.01393
106	Myst1	MYST histone acetyltransferase 1	II	135056031	1.38	1.000	0.01408
89	Gm166	predicted gene 166	II	134726602	1.78	1.000	0.01517
97	Orai3	ORAI calcium release-activated calcium modulator 3	II	134913329	1.83	1.000	0.01523
79	Zfp747	zinc finger protein 747	II	134516078	1.38	1.000	0.01591
56	Tmem219	RIKEN cDNA 2900092E17	II	134029685	1.34	1.000	0.01803
82	Zfp764	zinc finger protein 764	II	134547182	1.38	1.000	0.01973
7	Nsmce1-001	non-SMC element 1 homolog	I	132611154	1.23	1.000	0.02372
39	Ccdc101	coiled-coil domain containing 101	II	133792823	1.45	1.000	0.02725
109	Fus	malignant liposarcoma gene	II	135110971	1.34	1.000	0.02826
135	Klf2	Kruppel-like factor 3	III	74842932	-1.39	1.000	0.03117
134	Flt1	vascular endothelial growth factor receptor-1	III	148373180	1.40	1.000	0.03128

21	Xpo6	exportin 6	II	133245237	1.25	1.000	0.03363
98	Setd1a	SET domain containing 1A	II	134920184	1.25	1.000	0.03662
28	Rabep2	rabaptin	II	133572273	1.43	1.000	0.03930
67	Qprt-003	quinolinate phosphoribosyltransferase	II	134250628	2.11	1.000	0.04002
102	Zfp668	zinc finger protein 668	II	135008684	1.37	1.000	0.04010
104	BC039632	protease, serine, 53	II	135029355	1.46	1.000	0.04124
32	Atxn2l	ceroid lipofuscinosis, neuronal 3	II	133635224	1.22	1.000	0.04125
146	p27	cyclin-dependent kinase inhibitor p27	IV	134870419	1.23	1.000	0.04141
71	Sept1	septin 1	II	134357961	1.34	1.000	0.04567
33	Eif3c	eukaryotic translation initiation factor 3	II	133690426	1.20	1.000	0.04775
101	Stx4a	syntaxin 4A	II	134967808	1.30	1.000	0.04852

Expression assay by NanoString nCounter. 3 RNA samples of pia at each time point for each strain, with each sample composing of ≥ 8 embryos from ≥ 2 litters (18 samples, ~200 embryos). Transcript number for each gene normalized to mean transcript number for 6 housekeeping genes: *βactin, Gapdh, Tubb5, Hprt1, Ppia, Tbp.*
Gene number same as in Figure 6 and Table 1.

† **I**, genes within the EMMA region; **II**, gene s in 95% CI of Chr 7 QTL; **III**, angiogenesis-related genes located elsewhere in genome; **IV**, proliferation-related genes located elsewhere in genome.

‡ Fold change for BALB/c vs B6 if positive and B6 vs BALB/c if negative.

§ Bonferroni-adjusted p-values (p-value÷ 50) from t-tests independent of embryonic days (1 gene <0.05)

‖ p values from t tests independent of embryonic days, without Bonferroni adjustment (49 genes < 0.05).
