# Supplementary material for: Genetic Dissection of the Canq1 Locus Governing Variation in Extent of the Collateral Circulation
Source: PLoS One. 2012 Mar 6;7(3):e31910. doi: 10.1371/journal.pone.0031910 (PMC3295810; doi:10.1371/journal.pone.0031910)
Supplement: Table S5 — T test analysis of expression for 150 genes. Expression assay by NanoString nCounter. 3 RNA samples of pia at each time point for each strain, with each sample composing of ≥8 embryos from ≥2 litters (18 samples, ∼200 embryos). Transcript number for each gene normalized to mean transcript number for 6 housekeeping genes: βactin, Gapdh, Tubb5, Hprt1, Ppia, Tbp. • Gene number same as in Figure 6 and Table 1. † I, genes within the EMMA region; II, gene s in 95% CI of Chr 7 QTL; III, angiogenesis-related genes located elsewhere in genome; IV, proliferation-related genes located elsewhere in genome. ‡ Fold change for BALB/c vs B6 if positive and B6 vs BALB/c if negative. § Bonferroni-adjusted p-values (p-value÷ 50) from t-tests independent of embryonic days (1 gene <0.05). ∥ p values from t tests independent of embryonic days, without Bonferroni adjustment (49 genes <0.05). (PDF) [file pone.0031910.s013.pdf]

Table S5. T test analysis of expression for 150 genes

Gene* number	Name	Accession number	Gene[†] Group	Start	Fold[‡]	Bonadj[§]	P value[ll]
1	EG545999	XM_620562.3	I	132218849	1.01	1.000	0.86960
2	LOC670828	LOC670828.1	I	132362963	1.22	1.000	0.07918
3	4930533L02Rik	AK015957.1	I	132461873	-1.11	1.000	0.42103
4	4933440M02Rik	XR_035453.1	I	132473157	-1.11	1.000	0.57377
5	Jmjd5-001	ENSMUST00000033010.1	I	132588190	1.08	1.000	0.51411
6	Jmjd5-002	ENSMUST00000135129.1	I	132588190	-1.16	1.000	0.47913
7	Nsmce1-001	ENSMUST00000033006.1	I	132611154	1.23	1.000	0.02372
8	Nsmce1-002	ENSMUST00000138616.1	I	132611154	2.21	0.327	0.00218
9	Nsmce1-003	ENSMUST00000149289.1	I	132611154	4.35	0.166	0.00111
10	EG244214	XR_002216.2	I	132690783	1.13	1.000	0.46553
11	Il4ra-201	ENSMUST00000033004.1	I	132695785	-1.82	0.062	0.00041
12	Il21r-201	ENSMUST00000033000.1	I	132746983	-1.29	0.485	0.00323
13	Il21r-202	ENSMUST00000084605.1	I	132746983	-1.20	1.000	0.14259
14	Gtf3c1-201	ENSMUST00000055506.1	I	132784469	1.13	1.000	0.21177
15	Gtf3c1-202	ENSMUST00000106423.1	I	132784469	-1.12	1.000	0.30796
16	D430042O09Rik-204	ENSMUST00000122337.1	II	132851390	2.87	0.349	0.00232
17	D430042O09Rik-205	ENSMUST00000124223.1	II	132851390	-1.27	1.000	0.19248
18	D430042O09Rik-206	ENSMUST00000132204.1	II	132851390	-1.05	1.000	0.53792
19	D430042O09Rik-209	ENSMUST00000155059.1	II	132851390	2.43	0.539	0.00360
20	AC150648.2	NM_001101488.1	II	133024217	-1.63	1.000	0.23288

21	Xpo6	NM_028816.2	II	133245237	1.25	1.000	0.03363
22	Sbk1	NM_145587.2	II	133416133	-1.11	1.000	0.66163
23	AC135809.1	XM_001479519.1	II	133442948	1.03	1.000	0.70277
24	Lat	NM_010689.2	II	133507346	-1.17	1.000	0.28316
25	Spns1	NM_023712.2	II	133513574	-1.15	1.000	0.32032
26	Nfatc2ip	NM_010900.2	II	133526368	1.52	0.489	0.00326
27	Cd19	NM_009844.2	II	133552519	1.22	1.000	0.37917
28	Rabep2	NM_030566.2	II	133572273	1.43	1.000	0.03930
29	Atp2a1	NM_007504.2	II	133589372	1.07	1.000	0.62050
30	Sh2b1	NM_001081459.1	II	133610508	1.05	1.000	0.45434
31	Tufm	NM_172745.3	II	133630892	1.13	1.000	0.17644
32	Atxn2l	NM_183020.1	II	133635224	1.22	1.000	0.04125
33	Eif3c	NM_146200.1	II	133690426	1.20	1.000	0.04775
34	Cln3	NM_001146311.1	II	133714721	1.46	0.817	0.00545
35	Apob48r	NM_138310.1	II	133728456	-1.03	1.000	0.78160
36	Il27	NM_145636.1	II	133732524	-1.03	1.000	0.88211
37	Nupr1	NM_019738.1	II	133766763	1.09	1.000	0.77378
38	2510046G10Rik	ENSMUST00000084587.1	II	133792737	1.19	1.000	0.06737
39	Ccdc101	NM_029339.2	II	133792823	1.45	1.000	0.02725
40	Sult1a1	NM_133670.1	II	133816379	1.13	1.000	0.75620

41	Giyd2	NM_029420.3	II	133832982	1.60	0.681	0.00454
42	Bola2	NM_175103.3	II	133838915	1.21	1.000	0.05126
43	Coro1a	NM_009898.2	II	133843287	1.02	1.000	0.82613
44	Mapk3	NM_011952.2	II	133903115	1.06	1.000	0.49065
45	Gdpd3	NM_024228.2	II	133909928	1.42	1.000	0.06606
46	Ypel3	NM_026875.2	II	133920469	1.15	1.000	0.07221
47	Tbx6	NM_011538.2	II	133924997	2.06	0.150	0.00100
48	Ppp4c	NM_019674.3	II	133929421	1.05	1.000	0.60194
49	Aldoa	NM_007438.3	II	133938748	1.11	1.000	0.32887
50	Fam57b-201	ENSMUST00000079423.1	II	133963344	1.26	1.000	0.29276
51	Fam57b-202	ENSMUST00000098032.1	II	133963344	1.17	1.000	0.29742
52	4930451I11Rik	NM_183131.2	II	133973988	-1.26	1.000	0.22593
53	Doc2a	NM_010069.1	II	133990930	1.53	1.000	0.17702
54	Ino80e	NM_153580.1	II	133995094	3.01	0.474	0.00316
55	Taok2	NM_001163774.1	II	134009192	1.42	0.569	0.00380
56	Tmem219	NM_028389.1	II	134029685	1.34	1.000	0.01803
57	Kctd13	NM_172747.2	II	134072393	1.06	1.000	0.74609
58	Sez6l2	NM_144926.4	II	134085658	1.03	1.000	0.92376
59	Asphd1	NM_001039645.1	II	134089081	1.30	1.000	0.25334
60	Cdipt	NM_138754.3	II	134119902	1.09	1.000	0.34933

61	Mvp	NM_080638.2	II	134130382	-1.11	1.000	0.35167
62	2900092E17Rik	NM_030240.1	II	134144996	1.56	1.000	0.01063
63	Kif22	NM_145588.1	II	134171246	1.26	1.000	0.12636
64	AI467606	NM_178901.3	II	134234873	1.13	1.000	0.30448
65	Qprt-001	ENSMUST00000032912.1	II	134250628	-1.31	1.000	0.17939
66	Qprt-002	ENSMUST00000129332.1	II	134250628	1.40	1.000	0.08781
67	Qprt-003	ENSMUST00000142403.1	II	134250628	2.11	1.000	0.04002
68	Cd2bp2	NM_027353.3	II	134337200	1.16	1.000	0.07996
69	Tbc1d10b	NM_144522.5	II	134340979	-1.09	1.000	0.23995
70	Mylpf	NM_016754.5	II	134355122	1.03	1.000	0.89191
71	Sept1	NM_017461.2	II	134357961	1.34	1.000	0.04567
72	AC133494.1	NR_030674.1	II	134376338	2.29	0.868	0.00579
73	Zfp553	NM_146201.1	II	134376575	-1.09	1.000	0.48189
74	Zfp771	NM_177362.3	II	134388040	1.01	1.000	0.84065
75	Dctpp1	NM_023203.1	II	134400473	1.12	1.000	0.32510
76	Sephs2	NM_009266.3	II	134416075	1.02	1.000	0.78270
77	Itgal-003	ENSMUST00000120857.1	II	134439774	1.50	1.000	0.08231
78	Zfp768	NM_146202.1	II	134486312	1.10	1.000	0.31300
79	Zfp747	NM_175560.3	II	134516078	1.38	1.000	0.01591
80	9130019O22Rik	NM_030226.2	II	134525774	2.63	0.248	0.00166

81	AC133494.2	NM_198011.1	II	134527268	1.17	1.000	0.11350
82	Zfp764	NM_146203.3	II	134547182	1.38	1.000	0.01973
83	Zfp689	NM_175163.3	II	134587266	1.29	1.000	0.06462
84	Prr14	NM_145589.2	II	134603125	1.15	1.000	0.13184
85	Fbrs	NM_010183.1	II	134629170	1.11	1.000	0.15431
86	Srcap	XM_001480403.1	II	134655516	1.17	1.000	0.11975
87	1700008J07Rik	NR_024331.1	II	134655683	2.10	0.395	0.00263
88	Phkg2	NM_026888.3	II	134716854	1.46	0.554	0.00369
89	Gm166	NM_001033040.2	II	134726602	1.78	1.000	0.01517
90	Rnf40	NM_172281.1	II	134732281	1.11	1.000	0.13541
91	1700120K04Rik	NR_027915.1	II	134747592	2.11	0.104	0.00069
92	Zfp629	NM_177226.5	II	134750545	1.13	1.000	0.23763
93	Bcl7c	NM_009746.2	II	134806867	-1.17	1.000	0.05034
94	Ctf1	NM_007795.1	II	134856258	1.19	1.000	0.16982
95	Ctf2	NM_198858.1	II	134861620	1.03	1.000	0.86442
96	Fbxl19	NM_172748.2	II	134890289	1.18	1.000	0.25845
97	Orai3	NM_198424.3	II	134913329	1.83	1.000	0.01523
98	Setd1a	NM_178029.3	II	134920184	1.25	1.000	0.03662
99	Hsd3b7	NM_133943.2	II	134929122	1.11	1.000	0.50343
100	Stx1b	NM_024414.2	II	134947414	1.36	1.000	0.18455

101	Stx4a	NM_009294.3	II	134967808	1.30	1.000	0.04852
102	Zfp668	NM_146259.3	II	135008684	1.37	1.000	0.04010
103	Zfp646	NM_172749.4	II	135020310	1.22	1.000	0.05222
104	BC039632	NM_001081268.1	II	135029355	1.46	1.000	0.04124
105	Vkorc1	NM_178600.2	II	135029741	1.13	1.000	0.23264
106	Myst1	NM_026370.1	II	135056031	1.38	1.000	0.01408
107	Prss8	NM_133351.2	II	135069232	1.16	1.000	0.45531
108	Prss36	NM_001081374.1	II	135076152	1.14	1.000	0.24651
109	Fus	NM_139149.2	II	135110971	1.34	1.000	0.02826
110	B230325K18Rik	NM_176936.2	II	135126593	6.13	0.273	0.00182
111	Pycard	NM_023258.4	II	135135617	-2.24	0.006	0.00004
112	Trim72	NM_001079932.2	II	135147503	1.91	1.000	0.01393
113	Itgax	NM_021334.2	II	135273061	1.30	1.000	0.13166
114	Itgad	NM_001029872.1	II	135298292	-1.09	1.000	0.54568
115	Cox6a2	NM_009943.2	II	135349128	1.07	1.000	0.71821
116	9130023H24Rik	NM_177001.3	II	135379920	1.22	1.000	0.07621
117	Armc5	NM_146205.2	II	135381630	-1.14	1.000	0.12469
118	Tgfb1i1	NM_009365.2	II	135390385	4.19	0.549	0.00366
119	Slc5a2	NM_133254.3	II	135409171	1.13	1.000	0.58179
120	BC017158	NM_145590.1	II	135414893	1.33	1.000	0.00827

121	Rgs10	NM_026418.2	II	135517135	-1.16	1.000	0.27916
122	Tial1	NM_009383.2	II	135583291	1.58	0.893	0.00595
123	Bag3	NM_013863.4	II	135667130	-1.38	0.200	0.00134
124	Inpp5f	NM_178641.5	II	135754842	1.65	0.121	0.00081
125	Ctbp2	NM_001170744.1	II	140179304	1.05	1.000	0.58117
126	LOC100043248	XM_001479821.1	II	141616800	1.19	1.000	0.33198
127	Dock1	NM_001033420.2	II	141862370	1.77	0.615	0.00410
128	Angpt1	NM_009640.3	III	42256273	1.04	1.000	0.80124
129	Angpt2	NM_007426.3	III	18690263	1.07	1.000	0.76134
130	Clic4	NM_013885.2	III	134769884	-1.10	1.000	0.26607
131	Dll4	NM_019454.2	III	119151520	1.13	1.000	0.37811
132	Ephrinb2	NM_010111.5	III	8617434	-1.03	1.000	0.79252
133	Flk1	NM_010612.2	III	76328852	1.18	1.000	0.14194
134	Flt1	NM_010228.3	III	148373180	1.40	1.000	0.03128
135	Klf2	NM_008452.2	III	74842932	-1.39	1.000	0.03117
136	Klf4	NM_010637.3	III	55540015	-1.06	1.000	0.74389
137	Pdgfa	NM_008808.3	III	139451968	1.10	1.000	0.34480
138	Pdgfb	NM_011057.3	III	79826330	-1.08	1.000	0.56640
139	Tgfb1	NM_019919.2	III	75404869	1.10	1.000	0.35738
140	Vegfa188	NM_001025250.3	III	46153942	1.51	1.000	0.00772

141	Ampk	NM_001013367.3	IV	5093861	1.21	1.000	0.05706
142	Ki67	NM_001081117.2	IV	142881772	1.26	1.000	0.06787
143	Lkb1	NM_011492.3	IV	79578548	1.03	1.000	0.64946
144	p16INK4a	NM_009877.2	IV	88920377	1.07	1.000	0.68792
145	p21	NM_007669.4	IV	29227924	1.08	1.000	0.39289
146	p27	NM_009875.4	IV	134870419	1.23	1.000	0.04141
147	p53	NM_011640.1	IV	69393861	1.19	1.000	0.07964
148	Pcna	NM_011045.2	IV	132074898	1.05	1.000	0.57959
149	Sirt1	NM_019812.1	IV	62781753	1.19	1.000	0.11639
150	telomerase	NM_009354.1	IV	73764438	2.87	0.340	0.00227

Expression assay by NanoString nCounter. 3 RNA samples of pia at each time point for each strain, with each sample composing of ≥ 8 embryos from ≥ 2 litters (18 samples, ~200 embryos). Transcript number for each gene normalized to mean transcript number for 6 housekeeping genes: *βactin, Gapdh, Tubb5, Hprt1, Ppia, Tbp.*

•Gene number same as in Figure 6 and Table 1.

† **I**, genes within the EMMA region; **II**, gene s in 95% CI of Chr 7 QTL; **III**, angiogenesis-related genes located elsewhere in genome; **IV**, proliferation-related genes located elsewhere in genome.

‡ Fold change for BALB/c vs B6 if positive and B6 vs BALB/c if negative.

§ Bonferroni-adjusted p-values (p-value÷ 50) from t-tests independent of embryonic days (1 gene <0.05)

|| p values from t tests independent of embryonic days, without Bonferroni adjustment (49 genes <0.05).
