# Supplementary material for: Genetic Dissection of the Canq1 Locus Governing Variation in Extent of the Collateral Circulation
Source: PLoS One. 2012 Mar 6;7(3):e31910. doi: 10.1371/journal.pone.0031910 (PMC3295810; doi:10.1371/journal.pone.0031910)
Supplement: Materials and Methods S1 — (PDF) [file pone.0031910.s014.pdf]

Supplemental Information

Dissection of Chromosome 7 *Canq1* Locus Governing Variation in Extent of the Native Collateral Circulation

Shiliang Wang, Hua Zhang, Timothy Wiltshire, Robert Sealock and James Faber

Supplemental Materials

Additional Materials and Methods

Table S1 (qRT-PCR probes)

Table S2 (ANOVA analysis of 150 transcripts)

Table S3 (significant genes by t-test analysis)

Table S4 (t-test analysis of 150 genes)

Table S5 (Differential blood count)

Figures S1-S8

Material and Methods

Phenotyping

Mice were phenotyped for collateral number, diameter and cerebral artery tree territories. The circulation was cleared and the pial circulation overlying the dorsal cerebral cortex where the leptomeningeal collaterals reside was exposed. Arterial vessels were then maximally dilated, filled with a casting agent and fixed with paraformaldehyde. All collaterals interconnecting the anterior (ACA) and middle (MCA) cerebral artery trees in both hemispheres were counted and expressed as the average for one hemisphere. Lumen diameter of each collateral was measured at its midpoint from digitized images and averaged (Leica MZ16FA, Bannockburn, IL). Cortical territories supplied by the MCA, ACA and PCA trees of both hemispheres were determined morphometrically and averaged. Where possible, the investigator was blinded to genotype or group in this and other procedures below.

Middle Cerebral Artery Occlusion and Measurement of Infarct Volume

The MCA trunk was exposed midway between the zygomatic and temporal bones, cauterized and transected. 24 hours later the brain was sliced into 1 mm coronal sections that were stained with 2% triphenyltetrazolium chloride (TTC). Infarct volumes measured morphometrically were the sum of the cerebral cortical volume devoid of TTC in each section and expressed as a percent of total cortex volume.
