# Supplementary material for: Genetic Dissection of the Canq1 Locus Governing Variation in Extent of the Collateral Circulation
Source: PLoS One. 2012 Mar 6;7(3):e31910. doi: 10.1371/journal.pone.0031910 (PMC3295810; doi:10.1371/journal.pone.0031910)

Figure S2. Heatmap of known and imputed SNPs within the EMMA region for 74 strains. Abscissa, physical locations of selected SNPs across the EMMA region ($p=2.2x10^{-5}$). Ordinate, 74 inbred strains. 15 strains at bottom (bracket) arranged from least-to-most collateral number, per Zhang et al [9]. Each vertical line represents a SNP (blue, SNP same as B6; green, different from B6; blank, genotype unknown).

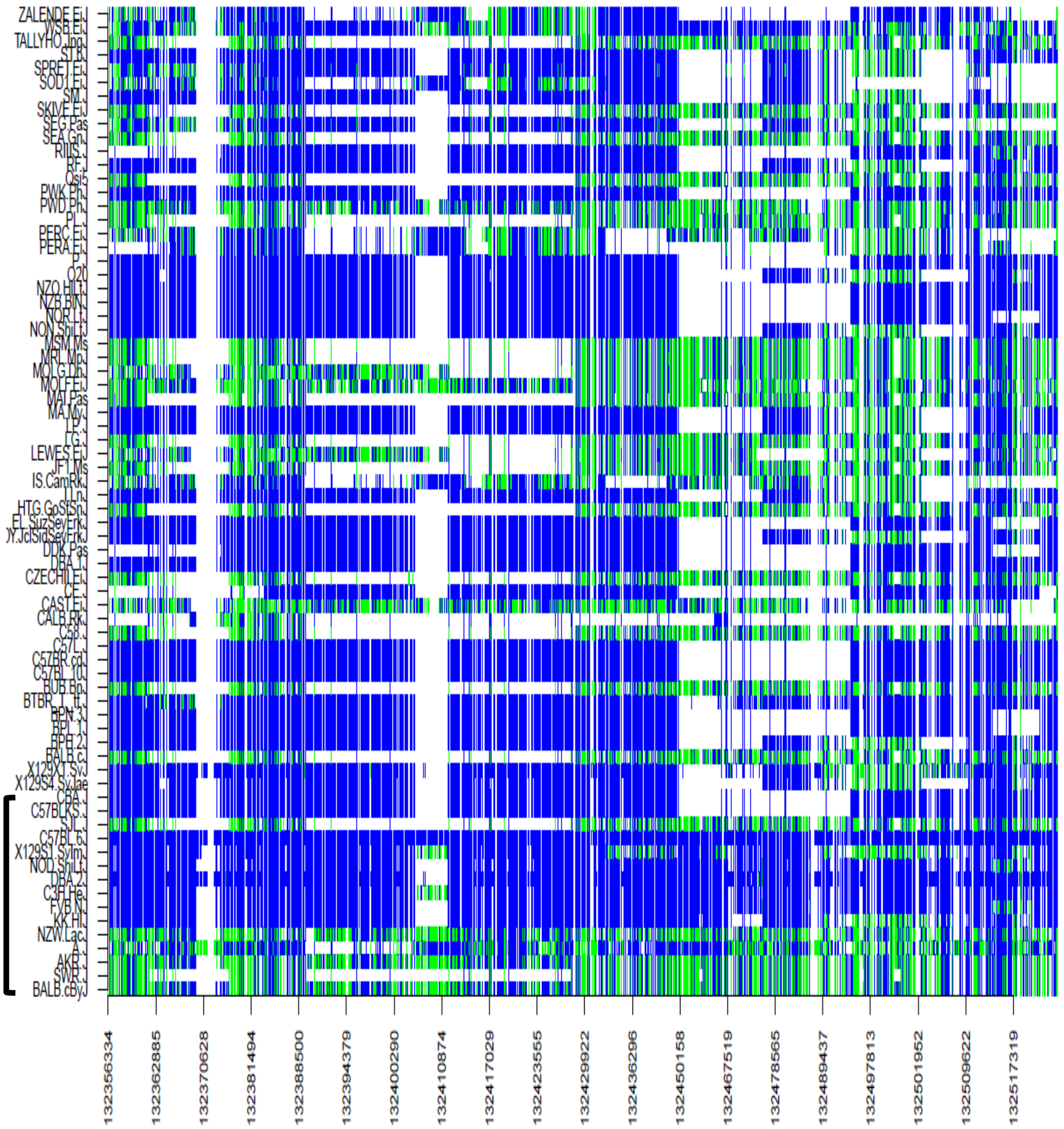

Supplement: Figure S2 — Heatmap of known and imputed SNPs within the EMMA region for 74 strains. Abscissa, physical locations of selected SNPs across the EMMA region (p = 2.2×10^−5^). Ordinate, 74 inbred strains. 15 strains at bottom (bracket) arranged from least-to-most collateral number, per Zhang et al. [9]. Each vertical line represents a SNP (blue, SNP same as B6; green, different from B6; blank, genotype unknown). (PDF) [file pone.0031910.s002.pdf]
